# Supplementary material for: AAV8 Gene Therapy for Crigler-Najjar Syndrome in Macaques Elicited Transgene T Cell Responses That Are Resident to the Liver
Source: Mol Ther Methods Clin Dev. 2018 Dec 5;11:191–201. doi: 10.1016/j.omtm.2018.10.012 (PMC6282099; doi:10.1016/j.omtm.2018.10.012)
Supplement: Document S1. Figures S1–S5 and Table S1 [file mmc1.pdf]

OMTM, Volume *11*

Supplemental Information

AAV8 Gene Therapy for Crigler-Najjar Syndrome in Macaques Elicited Transgene T Cell Responses That Are Resident to the Liver

Jenny A. Greig, Roberto Calcedo, Leticia Kuri-Cervantes, Jayme M.L. Nordin, Jessica Albrecht, Erin Bote, Tamara Goode, Edward A. Chroscinski, Peter Bell, Laura K. Richman, Michael R. Betts, and James M. Wilson

SUPPLEMENTAL INFORMATION

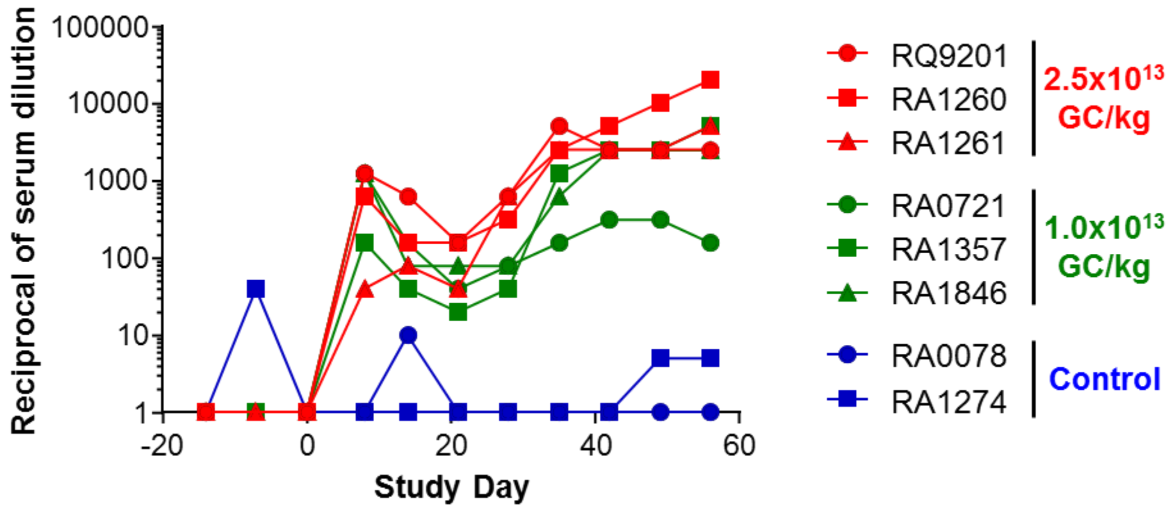

Figure S1. NAb levels in rhesus macaques administered with high or low dose of AAV8.TBG.hUGT1A1co compared to vehicle.
Rhesus macaques received an IV injection with $1.0x10^{13}$ GC/kg or $2.5x10^{13}$ GC/kg of AAV8.TBG.hUGT1A1co, or vehicle as a control. NAb levels to the AAV8 capsid were measured in serum samples taken throughout the study. The limit of detection of the assay is 5 (reciprocal of serum dilution).

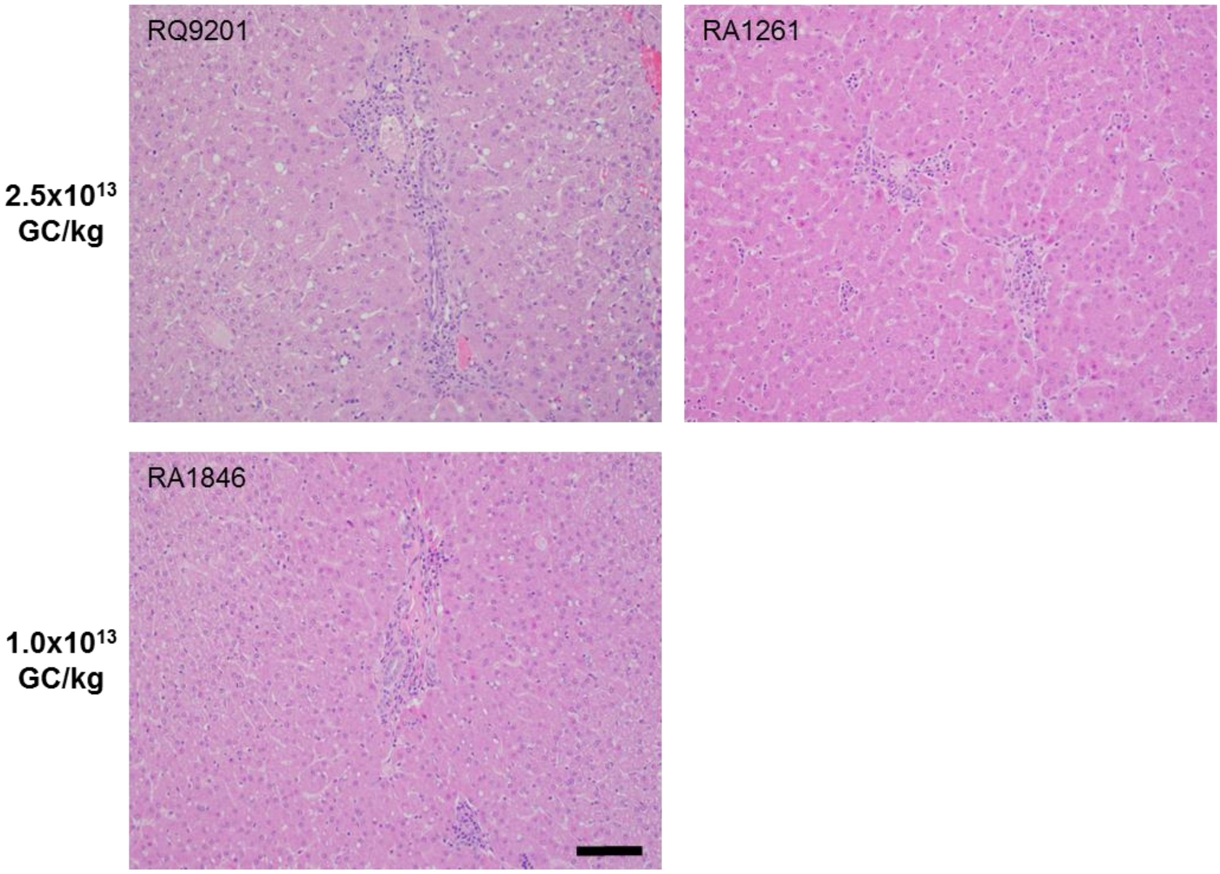

Figure S2. Liver histopathology in vector-administered macaques.
Representative images were taken from three macaques administered with vector to show areas with minimal to mild mononuclear cell infiltrate. Scale bar = 200 µm.

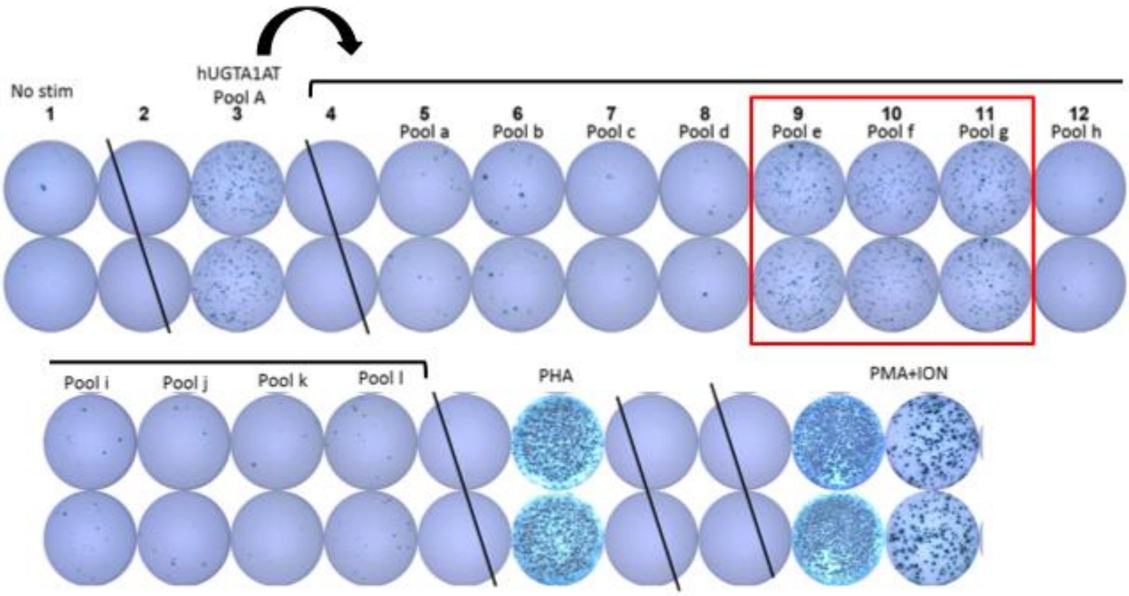

hUGT1A1 Pool A Sub-pools:

Pool	a	b	c	d	e	f
g	1	2	3	4	5	6
h	7	8	9	10	11	12
I	13	14	15	16	17	18
j	19	20	21	22	23	24
k	25	26	27	28	29	30
l	31	32	33	34	35	

5 : GPVVSHAGKILLIPV
6 : HAGKILLIPVDGSHW

Figure S3. Epitope mapping within the hUGT1A1 peptide pool A.
IFN-γ ELISPOT assay using PBMCs from animal RQ9201 that were stimulated with the hUGT1A1 peptide pool A and the matrix of sub-pools (a-l) generated from hUGT1A1 pool A. Positive responses were seen in sub-pools e, f, and g. The immunodominant epitope in the human sequence is shown in red. Phytohemagglutinin (PHA) or a phorbol 12-myristate 13-acetate and ionomycin (PMA+ION) at two cell concentration were used as stimuli for positive controls.

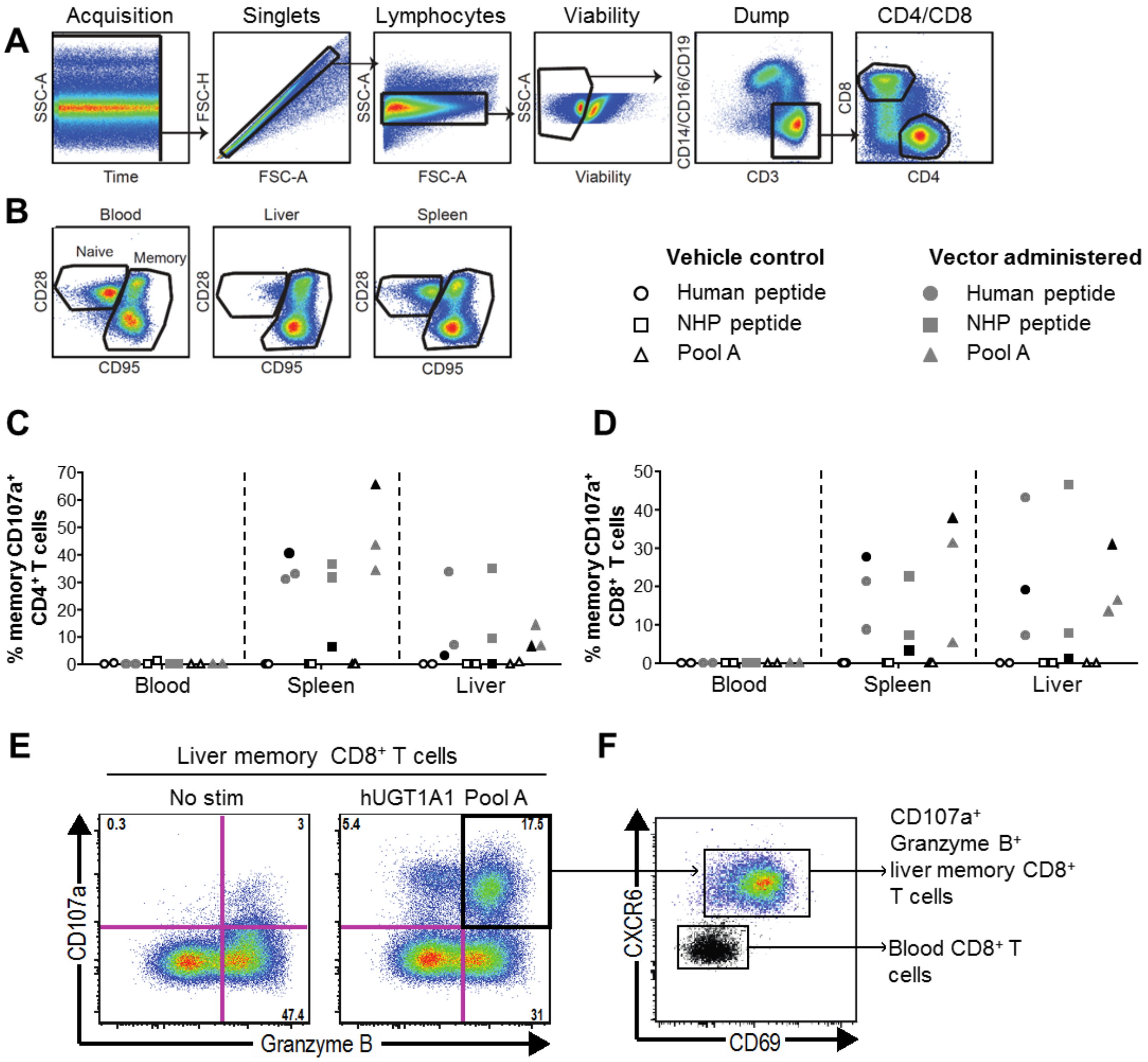

Figure S4. **Peptide-specific memory T-cell responses to AAV8.TBG.hUGT1A1co in blood and secondary lymphoid organs.**
(A) Representative plots of the gating strategy in peripheral blood where single viable lymphocytes were selected. $CD4^+$ or $CD8^+$ T cells were then selected from $CD3^+$ T cells, excluding monocytes, natural killer cells, and B cells. Naïve T cells ($CD28^+$, $CD95^-$) were gated out to further analyze peptide-specific responses within memory T cells. (B) Examples of the distribution of memory and naïve $CD8^+$ T cells in blood, liver, and spleen are shown. Lymphocytes from vehicle- (open symbols) or vector-administered rhesus macaques (closed symbols; low dose, gray; high dose, black) were cultured for nine hours in the presence of hUGT1A1 immunodominant epitope (circles, HAGKILLIPV), NHP UGT1A1 self-epitope (squares, HAGKMLLIPV), or hUGT1A1 pool A (triangles). Peptide-specific T-cell responses were measured in $CD107a^+$, $CD4^+$ memory T cells (C) and $CD107a^+$, $CD8^+$ memory T cells (D) derived from blood, spleen, and liver taken at the time of necropsy. (E) Representative plots of peptide-specific responses in liver lymphocytes of an animal administered with vector. The numbers inside the quadrants indicate percentages of memory $CD8^+$ T cells. (F) CXCR6/CD69 tissue-resident phenotype within peptide-specific $CD8^+$ T cells (color) in the liver and peripheral blood $CD8^+$ T cells (black).

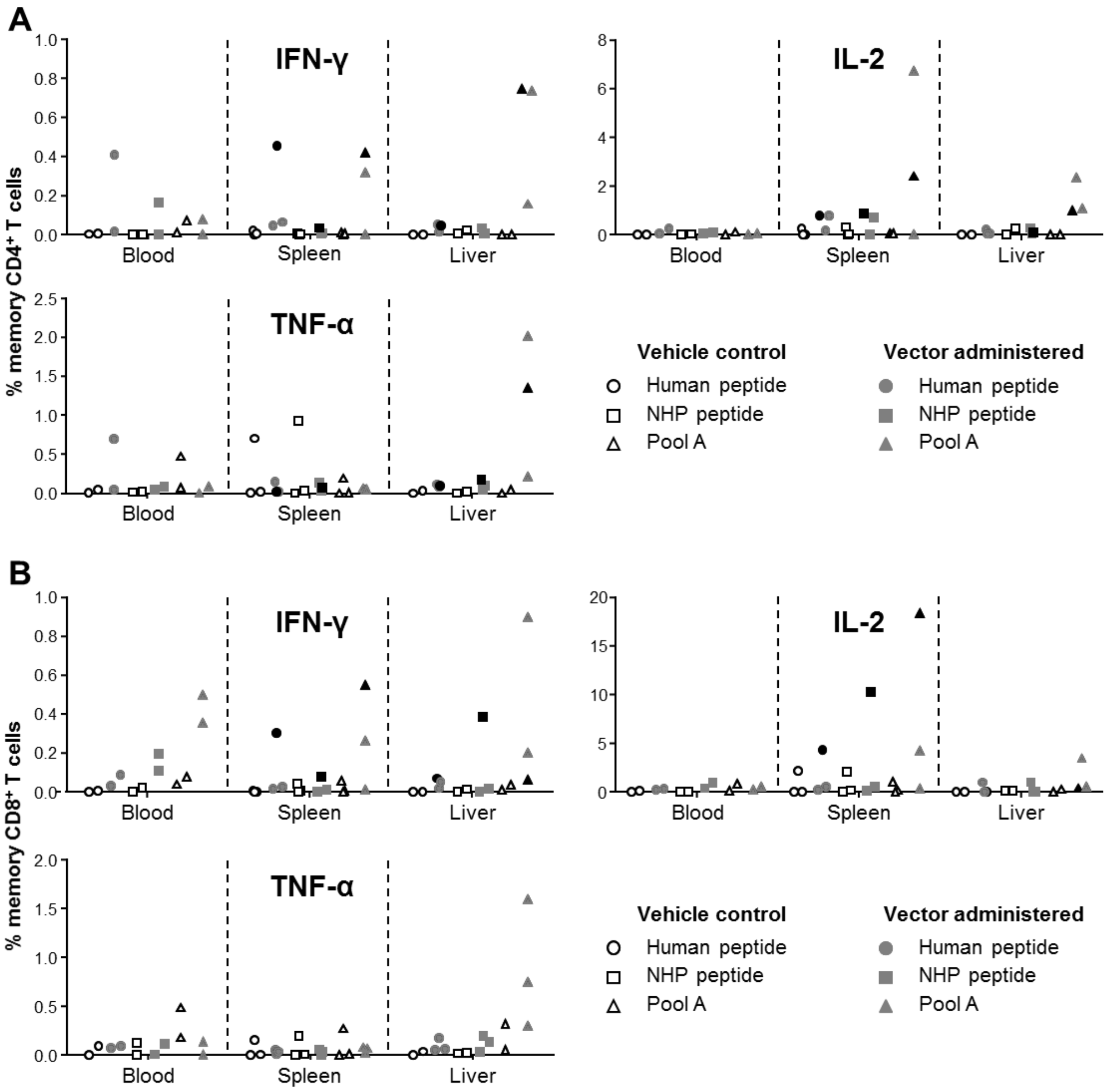

Figure S5. Flow cytometric analysis of lymphocytes isolated from peripheral blood and secondary lymphoid organs.

Peptide-specific production of IFN-γ, IL-2, and TNF-α in memory $CD4^+$ T cells (A) and $CD8^+$ T cells (B) from peripheral blood, spleen, and liver from vehicle- (open symbols) or vector-administered rhesus macaques (closed symbols; low dose, gray; high dose, black) were measured after nine hours of culture in response to stimulation with hUGT1A1 immunodominant epitope (circles, HAGKILLIPV), NHP UGT1A1 self-epitope (squares, HAGKMLLIPV), or hUGT1A1 pool A (triangles).

Tissues Collected For Histopathology		
Adrenal gland, left	Liver (left, middle, right, caudate)	Small intestine, duodenum
Adrenal gland, right	Lung, left	Small intestine, jejunum
Ascending aorta (proximal)	Lung, right	Small intestine, ileum (Peyers Patch)
Bone marrow, rib	Lacrimal gland	Spinal Cord
Brain	Lymph node, mandibular	Spleen
Cecum	Lymph node, mesenteric	Stomach
Cervix	Muscle, quadriceps femoris	Testes
Epididimides	Ovaries	Urinary Bladder
Esophagus	Pancreas	Uterus
Eye (left)	Prostate	Pituitary
Gall bladder	Rectum	Thymus
Heart	Salivary gland, mandibular	Thyroid gland (with parathyroid)
Kidney, left	Sciatic nerves	Trachea
Kidney, right	Seminal Vesicle	Vagina
Large intestine, colon	Skin with mammary	Gross lesions (if any)

Table S1. Tissues collected for histopathology.
Tissues collected for histopathological analysis at necropsy.
