# Supplementary material for: AAV8 Gene Therapy for Crigler-Najjar Syndrome in Macaques Elicited Transgene T Cell Responses That Are Resident to the Liver
Source: Mol Ther Methods Clin Dev. 2018 Dec 5;11:191–201. doi: 10.1016/j.omtm.2018.10.012 (PMC6282099; doi:10.1016/j.omtm.2018.10.012)
Supplement: Document S2. Article plus Supplemental Information [file mmc2.pdf]

Molecular Therapy
Methods & Clinical Development
Original Article

AAV8 Gene Therapy for Crigler-Najjar Syndrome in Macaques Elicited Transgene T Cell Responses That Are Resident to the Liver

Jenny A. Greig,[1,3] Roberto Calcedo,[1,3] Leticia Kuri-Cervantes,[2] Jayme M.L. Nordin,[1] Jessica Albrecht,[1] Erin Bote,[1] Tamara Goode,[1] Edward A. Chroscinski,[1] Peter Bell,[1] Laura K. Richman,[1] Michael R. Betts,[2] and James M. Wilson[1]

[1]Gene Therapy Program, Department of Medicine, University of Pennsylvania, Philadelphia, PA, USA; [2]Department of Microbiology, Perelman School of Medicine, University of Pennsylvania, Philadelphia, PA, USA

Systemic delivery of adeno-associated viral (AAV) vectors has been evaluated for the treatment of several liver diseases, including homozygous familial hypercholesterolemia, ornithine transcarbamylase deficiency, and hemophilia. Here, we evaluated this approach for the treatment of Crigler-Najjar syndrome. We administered wild-type rhesus macaques with 1.0 $\times$ 10^{13} or 2.5 $\times$ 10^{13} genome copies/kg of an AAV serotype 8 vector expressing a codon-optimized version of human uridine diphosphate glucuronosyl transferase 1A1 (UGT1A1) from a liver-specific promoter. We extensively studied vector biodistribution, transgene expression, and immune responses following vector administration. All rhesus macaques survived until their scheduled necropsy at day 56 and showed no clinical abnormalities during the course of the study. Macaques administered with either vector dose developed a T cell response to the AAV capsid and/or transgene. We mapped the immunodominant epitope in the human UGT1A1 sequence, and we found no correlation between peripheral and tissue-resident lymphocyte responses. Upon further investigation, we characterized CD107a$^+$, granzyme B$^+$, CD4$^+$, and CD8$^+$ transgene-specific cellular responses that were restricted to tissue-resident T cells. This study highlights the importance of studying immune responses at the vector transduction site and the limited usefulness of blood as a surrogate to evaluate tissue-restricted T cell responses.

INTRODUCTION

Liver gene replacement therapy has the potential to effectively treat, and even cure, a number of diseases resulting from the lack of a single gene product in hepatocytes. Systemic delivery of gene therapy vectors packaged with the adeno-associated viral (AAV) serotype 8 capsid results in extensive liver transduction and has been previously evaluated by our group for non-clinical efficacy in the treatment of homozygous familial hypercholesterolemia,[1–3] ornithine transcarbamylase deficiency,[4] and hemophilia.[5–7] We sought to evaluate the effectiveness of a similar approach for the treatment of Crigler-Najjar syndrome.[8]

Deficiency of the intracellular enzyme uridine diphosphate glucuronosyl transferase 1A1 (*UGT1A1*) results in hyperbilirubinemia and jaundice, characteristic of Crigler-Najjar syndrome type 1. The current medical management plan for this ultra-rare autosomal recessive disorder consists of phototherapy for >10 hr per day starting at birth to convert bilirubin by photoisomerization into water-soluble isomers. Decreasing circulating unconjugated bilirubin levels reduces the risk of severe, irreversible neurological damage (e.g., encephalopathy and kernicterus). To date, the only curative therapy is a liver transplant prior to the onset of neurological damage. The continuous synthesis of UGT1A1 by the liver following systemic delivery of a gene therapy vector expressing UGT1A1 has the potential to be a simpler and safer treatment than liver transplantation.

The purpose of this study was to evaluate the potential toxicity and tolerability of our clinical candidate vector for expression of a codon-optimized version of human UGT1A1 (hUGT1A1co) following intravenous (i.v.) administration in rhesus macaques. As there is no large-animal model of Crigler-Najjar syndrome, we used wild-type rhesus macaques that have endogenous expression of rhesus UGT1A1 in the liver. With this limitation, we conducted a non-clinical pharmacology and toxicology study where rhesus macaques received an infusion of one of two doses of AAV vector expressing hUGT1A1co from the thyroxine-binding globulin (TBG) promoter (AAV8.TBG.hUGT1A1co) into a peripheral vein; an additional cohort of animals received an infusion of vehicle only. By evaluating our clinical candidate vector in a large-animal model, we were able to extensively study vector biodistribution, transgene expression and immune responses following vector administration. Recent reports have shown the relevance of CD8$^+$ T cell responses in non-lymphoid tissues, specifically by a newly described tissue-resident memory

Received 12 October 2018; accepted 25 October 2018;
https://doi.org/10.1016/j.omtm.2018.10.012.

[3]These authors contributed equally to this work.

Correspondence: James M. Wilson, Department of Microbiology, Perelman School of Medicine, University of Pennsylvania, 125 South 31st Street, Suite 1200, Philadelphia, PA 19104, USA.
E-mail: wilsonjm@upenn.edu

This is an open access article under the CC BY license (http://creativecommons.org/licenses/by/4.0/).

subset.[9–12] Tissue-resident memory CD8$^+$ T cells are functionally, phenotypically, and transcriptomically distinct from peripheral memory CD8$^+$ T cells.[13,14] Thus, we analyzed immune responses in blood and secondary lymphoid tissues, and identified a transgene-specific cellular response restricted to tissue-resident T cells.

RESULTS

Study Design and Overall Clinical Findings

To evaluate the potential toxicity and tolerability of the AAV8.TBG.hUGT1A1co vector, we examined two doses: 1.0×10^{13} and 2.5×10^{13} genome copies (GC)/kg. For each dose, two male and one female rhesus macaques received vector via i.v. administration. In addition, one male and one female rhesus macaque received an i.v. dose with vehicle control. Throughout the study, we observed the rhesus macaques daily and visually examined them each time they were anesthetized. Prior to the initiation of the study, we screened all rhesus macaques for neutralizing antibodies (NAbs) to the AAV8 capsid, as previously described.[15] All eight animals selected for the study were AAV8 seronegative (NAb titer <1:5). In addition, we monitored the AAV8 NAb response throughout the study. During the in-life phase of the study, we monitored the macaques for immune responses to the AAV8 capsid and the hUGT1A1 transgene by interferon gamma enzyme-linked immunospot (IFN-γ ELISPOT) of blood samples collected on days 0, 14, 28, 42, and 56. In addition, the contract facility Antech GLP analyzed changes in blood chemistries, cell profiles, and coagulation parameters of the animals every other week throughout the study. We performed a laparotomy procedure on day 28 to isolate liver tissue for vector biodistribution and expression analysis. All rhesus macaques survived until their scheduled necropsy at day 56, with no clinical abnormalities during the course of the study. At the time of necropsy, we isolated lymphocytes from liver, spleen, and bone marrow to evaluate tissue-specific immune responses. In addition, we collected tissue samples for histopathology (Table S1).

Time Course of Liver Transaminases, AAV8 NAbs, and T Cell Immune Responses in Rhesus Macaques

Rhesus macaques administered with vehicle control had no elevations in the liver transaminases aspartate aminotransferase (AST) and alanine aminotransferase (ALT) even after the liver biopsy procedure performed on day 28 (Figure 1, red arrow). Animals administered with the low dose (1.0×10^{13} GC/kg) of AAV8 vector had elevations in ALT and AST levels, but these were not statistically significant when compared to the vehicle control group. Animals administered with the high dose (2.5×10^{13} GC/kg) of AAV8 vector had a statistically significant difference in AST values when compared to the control group (p value = 0.010, linear mixed-effect modeling).

Unsurprisingly, we detected no IFN-γ ELISPOT-positive responses to either the AAV8 capsid or the hUGT1A1 transgene in the two animals that were administered with the vehicle control. Macaques administered with both low and high doses of AAV8.TBG.hUGT1A1co vector developed a T cell response to the AAV capsid and/or transgene (Figure 1, bar graphs). The transgene immune responses could be stratified into one of three dose-independent categories: (1) positive response in the blood only (RA1846 and RA1261), (2) positive response in tissues only (RA1357 and RA1260), or (3) positive response in both the blood and tissues (RA0721 and RQ9201). Macaques RA1846 and RA1261, administered with the low and high vector doses, respectively, showed positive responses only against the hUGT1A1 transgene and only in blood peripheral blood mononuclear cells (PBMCs). Unfortunately, liver lymphocytes were not available for RA1261 for analysis of the transgene-specific response, and only responses to the AAV8 capsid could be determined (Figure 1). Interestingly, RA1846 had a T cell response to the hUGT1A1 transgene even before vector administration (day 0; Figure 1). This pre-existing immune response may be explained by the presence of a shared epitope between the hUGT1A1 protein and a foreign epitope.

We detected IFN-γ ELISPOT-positive responses to hUGT1A1 in the liver of animals RA0721 and RA1357 (low vector dose) and RQ9201 and RA1260 (high vector dose) (Figure 1). Animal RQ9201 showed a similar response in spleen and bone marrow. The difference between these four macaques was the ability to detect transgene-specific positive responses in PBMCs, which we monitored throughout the in-life phase of the study. RA0721 and RQ9201 had positive responses in both the blood and tissues to the hUGT1A1 transgene, indicating that blood may not be predictive of a potential immune response in tissues. However, macaques RA1357 and RA1260 did not show any indication of an immune response to the transgene in blood; in these animals, we only detected a response in liver lymphocytes isolated at necropsy. This effect is not a function of vector dose, as one macaque from each dose group falls into each of the categories described above.

We detected T cell responses to the AAV8 capsid in blood (day 42) and liver of animal RA0721, in the liver of animal RQ9201, and in blood (day 56) of animal RA1260 (Figure 1). T cell responses to the AAV8 capsid were up to 10-fold lower in magnitude than the T cell responses to the hUGT1A1 transgene, and they did not persist over time. We did not find a correlation between ALT and AST elevations and positive IFN-γ ELISPOT responses to the AAV8 capsid or to the hUGT1A1 transgene in blood or tissue-resident T cells.

All animals had NAb titers of <1:5 prior to initiation of the study. Animal RA1274 was seronegative at day −14 and day 0 prior to administration of the vehicle control (Figure S1). However, this animal had an AAV8 NAb titer of 1:40 on day −7, and both vehicle-control-injected animals remained seronegative or had titers of 1:5 throughout the study (Figure S1). Natural fluctuations in AAV8 NAb titers in non-injected animals have been previously described.[16] Following vector administration, all animals developed an AAV8 NAb response, where titers increased from <1:5 to 1:40–1:1,280 (Figure S1). There was no dose-dependent effect of vector on NAb titer, as there was a similar and overlapping spread of NAb titers at both doses.

Liver Histopathological Findings

At the time of necropsy, we noted gross observations on the surface of the liver of two macaques (RQ9201 and RA1261—both administered

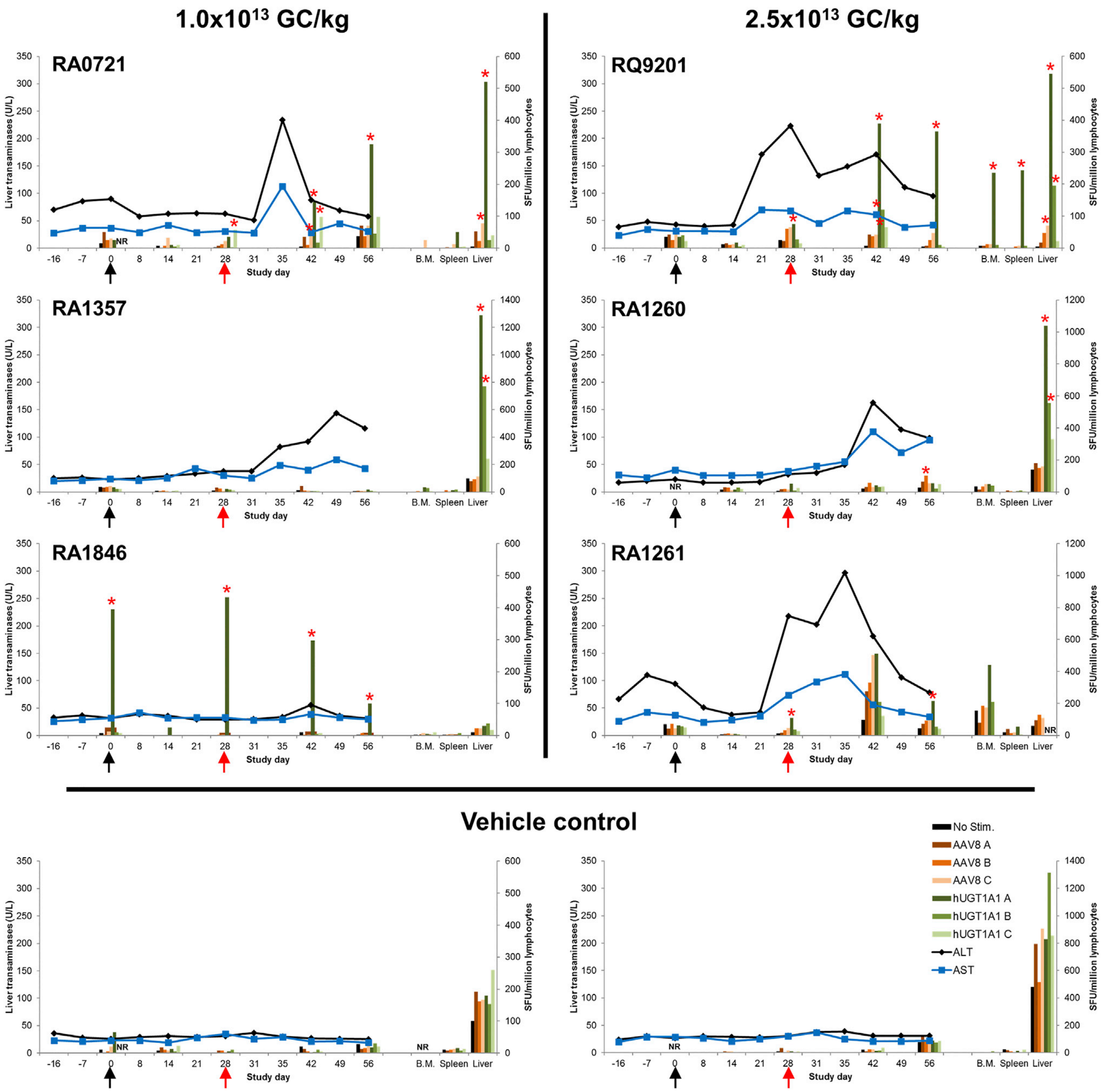

Figure 1. Time Course of Liver Transaminases and T Cell Immune Responses in Rhesus Macaques Administered with Low or High Dose of AAV8.TBG.hUGT1A1co Compared to Vehicle

Rhesus macaques received an i.v. injection with 1.0×10^{13} GC/kg or 2.5×10^{13} GC/kg of AAV8.TBG.hUGT1A1co or vehicle as a control. Liver transaminases (AST and ALT) were measured in serum samples taken throughout the study (lines). Peripheral T cell responses to the AAV8 capsid and the hUGT1A1 transgene were measured by IFN-γ ELISPOT (bars) throughout the study using peptide libraries specific for the AAV8 capsid (pools A, B, and C) and the hUGT1A1 transgene (pools A, B, and C). IFN-γ ELISPOT assays were also performed on lymphocytes isolated from bone marrow (B.M.), spleen, and liver at the time of necropsy (day 56 post-vector-administration). Asterisks indicate a T cell response that met the positive response criteria, >55 spot-forming units per 10^6 cells when stimulated with antigen and three times greater than the medium negative control value (No Stim.). The black arrows indicate the time of vector administration on study day 0, and the red arrows indicate the time of liver biopsy on day 28 post-vector-administration. NR, no lymphocytes available for analysis.

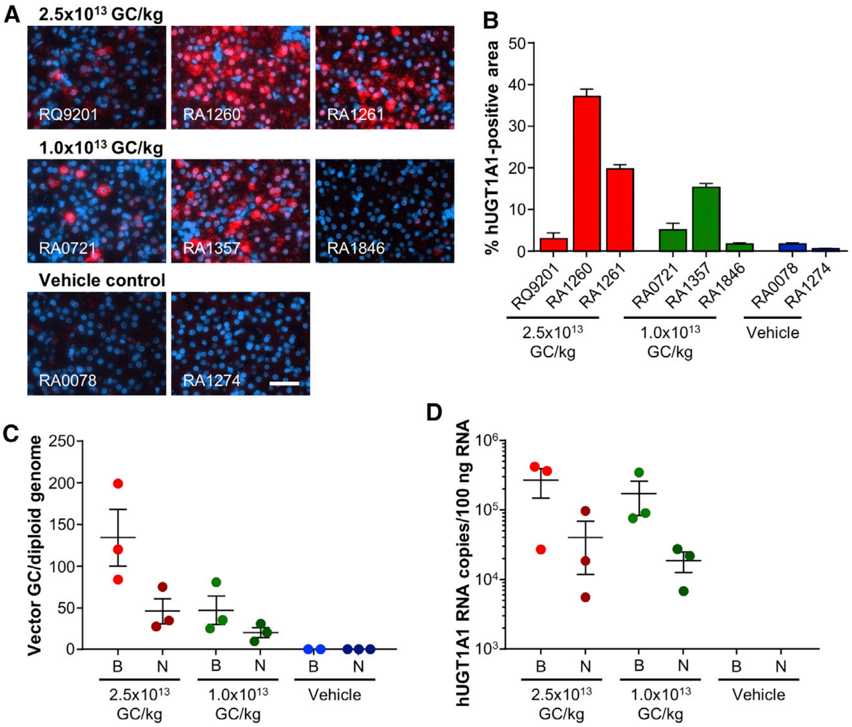

Figure 2. Evaluation of Liver hUGT1A1 RNA Levels and Vector GC

(A) ISH was performed on liver biopsy samples (day 28 post-vector-administration) to evaluate the expression of hUGT1A1 (red staining). The probe is specific for the hUGT1A1co sequence and avoids cross-hybridization with endogenous rhesus macaque RNA. Counterstaining was with DAPI (blue). Scale bar, 50 μm. (B) The percentage of the positive area by ISH was quantified from five images; values are presented as mean ± SEM. (C) DNA and (D) RNA were extracted from liver samples taken at liver biopsy and at necropsy on day 56 post-vector-administration. DNA and RNA levels were calculated for left, middle, and right lobes of the liver. The average value for each NHP is presented with the mean ± SE of the group. B, biopsy; N, necropsy.

with the high dose of vector). In both instances, the corresponding histologic findings were consistent with a reaction to the liver biopsy procedure on day 28 and the gelfoam used for hemostasis. All vector-administered macaques had minimal to mild mononuclear cell infiltration within the portal areas (representative images shown in Figure S2); one macaque administered with the low vector dose (RA0721) showed minimal Ito cell hyperplasia.

Detection of hUGT1A1co Expression in Rhesus Macaque Liver by *In Situ* Hybridization

Due to the use of wild-type rhesus macaques in this study and similarities between the rhesus and human UGT1A1 sequences, we were not able to evaluate the protein levels of the transgene. However, we did perform ViewRNA *in situ* hybridization (ISH) in order to detect hUGT1A1co RNA expression, since the codon-optimized transgene sequence was specific for the vector. We analyzed the liver sections using a fluorescent probe designed to avoid cross-hybridization with endogenous rhesus macaque RNA. Following biopsy on day 28, we evaluated sections of the liver using fluorescence microscopy with fast red staining, which indicated hepatocytes expressing hUGT1A1co (Figure 2A, red staining), and counterstained sections with DAPI (blue). We captured images from five liver sections and signal quantified each image using ImageJ software. There was a dose response in the hUGT1A1co-specific ISH staining, with little to no detectable background levels from the endogenous RNA (Figure 2B). The level of expression of hUGT1A1co RNA did not correlate with the presence of transgene-specific immune responses in the liver. We were not able to compare ISH performed on samples taken at the time of biopsy to those taken at necropsy due to an incompatible fixation treatment at necropsy.

Reduction in Liver Vector GCs and Transgene RNA from Biopsy to Necropsy

We evaluated liver tissues harvested by biopsy on day 28 and at necropsy on day 56 for vector biodistribution. DNA and RNA were extracted and quantified using qPCR that detected the vector poly(A) and a transgene-specific sequence, respectively. Unsurprisingly, we could not detect any vector GC (Figure 2C) or transgene RNA (Figure 2D) in macaques administered with the vehicle control. Animals that received the high vector dose showed, on average, higher levels of vector GC and transgene RNA in the liver, and all of these values decreased over the 28 days between tissue collection at biopsy and necropsy (Figures 2C and 2D). In the high-dose group, vector GC dropped 2.9-fold from an average of 134.3 GC per diploid genome at biopsy to 45.8 GC per diploid genome at necropsy (Figure 2C). By contrast, the low-dose group showed a decrease of only 2.3-fold, with vector GC decreasing from 47.1 to 20.3 GC per diploid genome (Figure 2C). Average transgene RNA levels decreased 6.7-fold and 9.2-fold for the high- and low-dose vector groups, respectively, from the biopsy time point to necropsy (Figure 2D). By the necropsy time point, there was not significant difference in vector GC or transgene RNA between the two vector doses (Student's t test, $p = 0.186$ and $p = 0.408$, respectively).

Epitope Mapping of the Transgene-Specific Immune Response

We further characterized the immune response to hUGT1A1 in order to identify the specific amino acid sequence (epitope) responsible for the T cell activation. The data allowed us to determine if the detected T cell response was directed against a region of UGT1A1 that was specific to the human transgene or that was shared across humans and macaques. The majority of the hUGT1A1-specific T cell responses in both the periphery and the liver were against pool A of the hUGT1A1 peptide library. Pool A

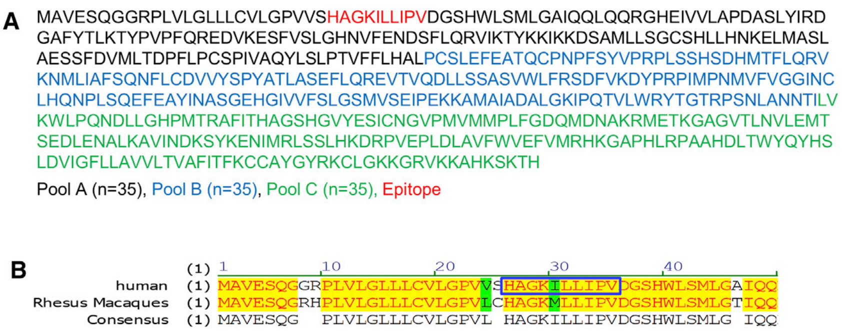

Figure 3. hUGT1A1 Epitope Location within Peptide Pool A

(A) The full amino acid sequence of the hUGT1A1 transgene is shown, indicating the peptides included in pool A (black), B (blue), and C (green), and the location of the immunodominant epitope (red). (B) Human and rhesus macaque UGT1A1 sequence alignment. The box indicates the immunodominant epitope in the human transgene sequence.

contains peptides from amino acids 1–175 of the hUGT1A1 protein sequence (Figure 3A). Approximately 95% of the amino acid sequence for UGT1A1 in humans and rhesus are conserved, and most of the species-specific differences in sequences occur within the first 175 amino acids (10 out of 25 amino acid differences). We performed epitope mapping to hUGT1A1 using samples deemed positive by IFN-γ ELISPOT. We developed a matrix of the peptides used in UGT1A1 pool A, in which each peptide was included in two sub-peptide pools (Figure S3). Sub-pools e, f, and g showed positive responses (Figure S3). Peptides 5 and 6 are the only peptides present in all three sub-pools. Therefore, we determined that the immunodominant epitope was the common sequence between peptides 5 and 6, HAGKILLIPV (shown in red in Figures 3A and S3). Interestingly, the HAGKILLIPV peptide contains one amino acid difference between the human and rhesus macaque sequences (Figure 3B), proving that the T cell response is directed against a region of UGT1A1 that is specific to the human transgene.

Tissue-Resident Memory Responses to the hUGT1A1 Transgene

Once we determined the epitope, we next sought to identify and characterize the T cell subset responsible for the T cell response to hUGT1A1. As the IFN-γ ELISPOT results indicated that the peripheral responses were not correlated to the tissue lymphocyte responses (Figure 1), we evaluated differences between the peripheral (blood taken at the time of necropsy) and tissue-resident T cell responses (liver and spleen). We stimulated lymphocytes from one high-dose animal, two low-dose animals, and both vehicle control animals with hUGT1A1 pool A, the immunodominant human epitope, and the non-human primate (NHP) sequence of the epitope. Then, we stained the lymphocytes for multiple markers, including lineage markers (CD3, CD4, and CD8), memory markers (CD28 and CD95), degranulation markers (CD107a and granzyme B), cytokine functionality (interleukin-2 [IL-2], tumor necrosis factor alpha [TNF-α], and IFN-γ), and liver homing and residency markers (CXCR6 and CD69, respectively), and analyzed the samples using polychromatic flow cytometry in an intracellular cytokine staining (ICS) assay. See Figures S4A and S4B for a representative gating strategy. The ICS analysis revealed that the T cell response to hUGT1A1 in tissues was dominated by CD107a degranulation factor and granzyme B (Figure S4E) expressed by $CD4^+$ (Figure 4A) and $CD8^+$ memory T cells (Figure 4B). This response was absent in blood PBMCs (Figure 4).

We observed these tissue-resident responses in all three AAV vector-injected animals (Figures 4C, 4D, S4C, and S4D). The magnitude of the response was as high as 68% of $CD107a^+$ and 13% of $CD107a^+$ and granzyme B^+ memory $CD4^+$ T cells in the spleen (Figures S4C and 4C, respectively), and as high as 49% of $CD107a^+$ and 25% of $CD107a^+$ and granzyme B^+ memory $CD8^+$ T cells in the liver (Figures S4D and 4D, respectively). Furthermore, hUGT1A1-specific T cells from the liver and spleen, but not from blood, co-expressed the residency and homing markers CD69 and CXCR6 (Figure S4F), thus confirming that these cells are indeed resident-memory T cells. Importantly, the dramatic response to the hUGT1A1 transgene did not trigger a cytotoxic T-lymphocyte response in the liver, as the only histopathological findings were minimal to mild infiltration. Cytokine responses to UGT1A1 were up to 100-fold lower than CD107a and granzyme B responses (Figure S5). IL-2 was the dominant cytokine and was present in both $CD4^+$ (Figure S5A) and $CD8^+$ (Figure S5B) T cells from tissues, but not blood (Figure S5). We detected IFN-γ and TNF-α at lower frequencies in both $CD4^+$ and $CD8^+$ T cells, in both tissues and blood (Figure S5).

Interestingly, the $CD107a^+$ and granzyme B^+ T cell response was specific to the human epitope of UGT1A1 in one animal (RQ9201) and cross-reactive to the NHP epitope in two animals (RA1357 and RA0721) (Figures 4C and 4D, 5, S4C, and S4D). In animals RA1357 and RA0721, AAV gene therapy resulted in the generation of cross-reactive T cells. We did not detect any $CD107a^+$ and granzyme B^+ or cytokine T cell responses to hUGT1A1 in blood or tissues from vehicle-control-injected animals. We performed major histocompatibility complex (MHC) I and II genotype analysis (Table 1) to evaluate if there was a correlation with the potential to generate T cells reactive to rhesus macaque UGT1A1. The macaques in this study were extremely diverse; only two macaques shared a similar haplotype (RA1357 and RA1274). Animals RA1357 and RA1274 were administered the low dose and vehicle control, respectively. Although T cells from RA1357 recognized the self-antigen, so did RA0721 even though it had no comparable haplotypes.

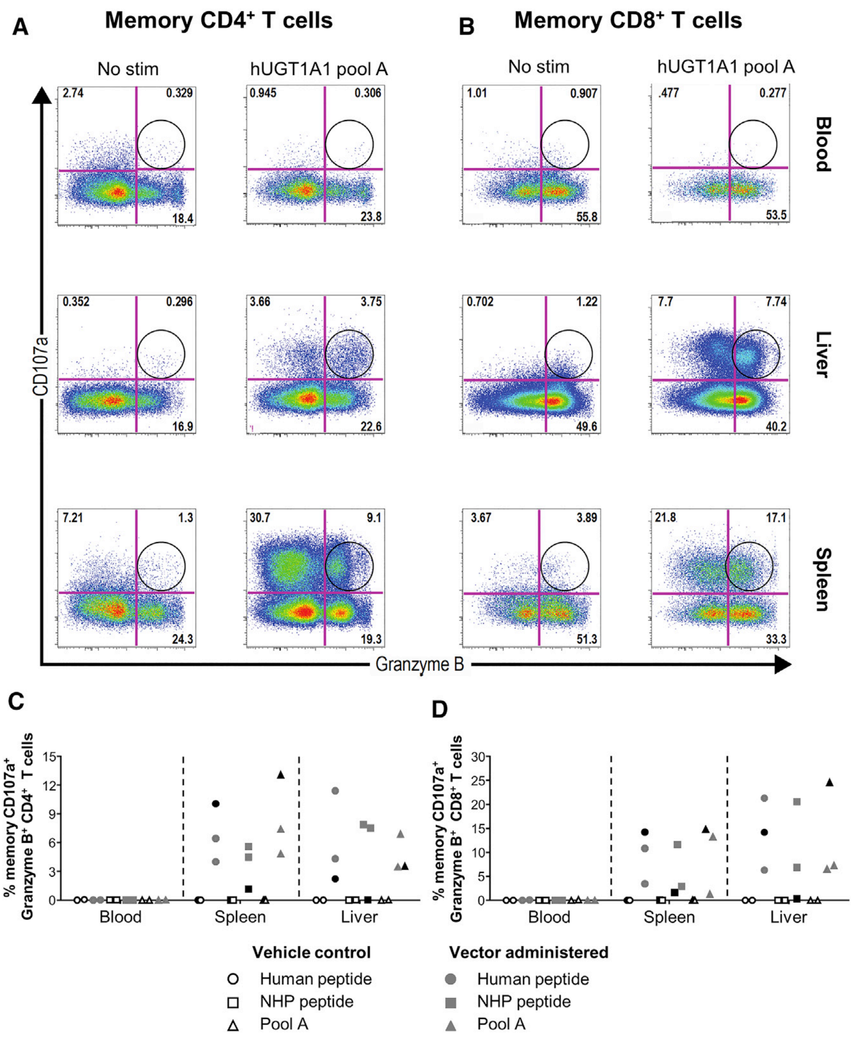

Figure 4. Evaluation of UGT1A1-Specific CD4+ and CD8+ T Cell Responses

Representative density plots of lymphocytes isolated from blood, spleen, and liver at necropsy from macaque RA0721. Lymphocytes were stimulated with UGT1A1 pool A or dimethyl sulfoxide (No stim). Surface expression of a degranulation factor (CD107a) and expression of granzyme B by memory $CD4^+$ (A) and memory $CD8^+$ (B) T cells were analyzed by an ICS assay. Memory T cells were defined as $CD95^+$, $CD28^+$ and $CD95^+$, $CD28^-$. Circles indicate the presence of $CD107a^+$, granzyme B^+ UGT1A1-specific T cells. Quantification of the T cell immune response of lymphocytes from vehicle- (open symbols) or vector-administered rhesus macaques (closed symbols; low dose, gray; high dose, black) after stimulation with hUGT1A1 immunodominant epitope (circles, HAGKILLIPV), NHP UGT1A1 self-epitope (squares, HAGKMLLIPV), or hUGT1A1 pool A (triangles). Peptide-specific T cell responses were measured in $CD107a^+$, granzyme B^+, $CD4^+$ memory T cells (C), and $CD107a^+$, granzyme B^+, $CD8^+$ memory T cells (D) derived from blood, spleen, and liver.

DISCUSSION

Liver gene therapy has the potential to treat and cure numerous metabolic disorders by expressing either a secreted or non-secreted transgene product. Here, we evaluated two doses of AAV8.TBG.hUGT1A1co, the clinical candidate vector for the treatment of Crigler-Najjar syndrome, for toxicity in rhesus macaques. We administered two doses (1.0×10^{13} GC/kg and 2.5×10^{13} GC/kg) that were lower than those used previously by our group and others for systemic delivery in large animals.[2,17–22] There was no clinical toxicity evident in this study, and gross pathology findings were limited to (1) a reaction to the liver biopsy procedure performed on day 28 and (2) minimal to mild infiltrates in the liver. We have shown complete and stable correction of hyperbilirubinemia in a mouse model of Crigler-Najjar syndrome at 2.5×10^{12} GC/kg, which is 10-fold lower than the upper dose studied in this NHP toxicity study, suggesting an attractive therapeutic window.[8,23]

Recent reports showed severe toxicity in NHPs with vectors similar to AAV9 at doses that were substantially higher than those in this study.[21,22] Following i.v. infusion of 2×10^{14} GC/kg of AAVhu68 expressing the human transgene for treatment of spinal muscular atrophy[21] or 7.5×10^{13} GC/kg of AAV-PHP.B expressing green fluorescent protein,[22] acute and severe hepatotoxicity led to the death of two animals on study days 4 and 5, respectively. The other animals dosed in these two studies were deemed clinically normal; although ALT and AST were elevated, they resolved with only minimal histopathological findings present following necropsy. The toxic events in these other high dose systemic AAV studies appeared to be triggered by an acute innate immune response. We did not observe this type of acute inflammatory response in this study, possibly reflecting the lower dose, a more favorable acute toxicity profile of AAV8 versus AAV9, or both. Nothing from our NHP toxicity studies suggests innate immune activation or systemic inflammation.

We specifically evaluated T cell immune responses to both the AAV8 capsid and the hUGT1A1 transgene. We found that i.v. administration of AAV8.TBG.hUGT1A1co vector induced a T cell response that was mainly directed against the hUGT1A1 transgene. The immune response to the transgene was expected, given that the AAV8.TBG.hUGT1A1co clinical candidate gene therapy vector expresses the human UGT1A1 protein sequence, and this study used rhesus macaques. Indeed, epitope mapping revealed that the T cell response to hUGT1A1 was directed to a region of the protein that contained one amino acid difference between the human and rhesus macaque sequences. One macaque from the high-dose group developed a T cell response that was specific to this region of the hUGT1A1 transgene. Two macaques from the low-dose group developed a T cell

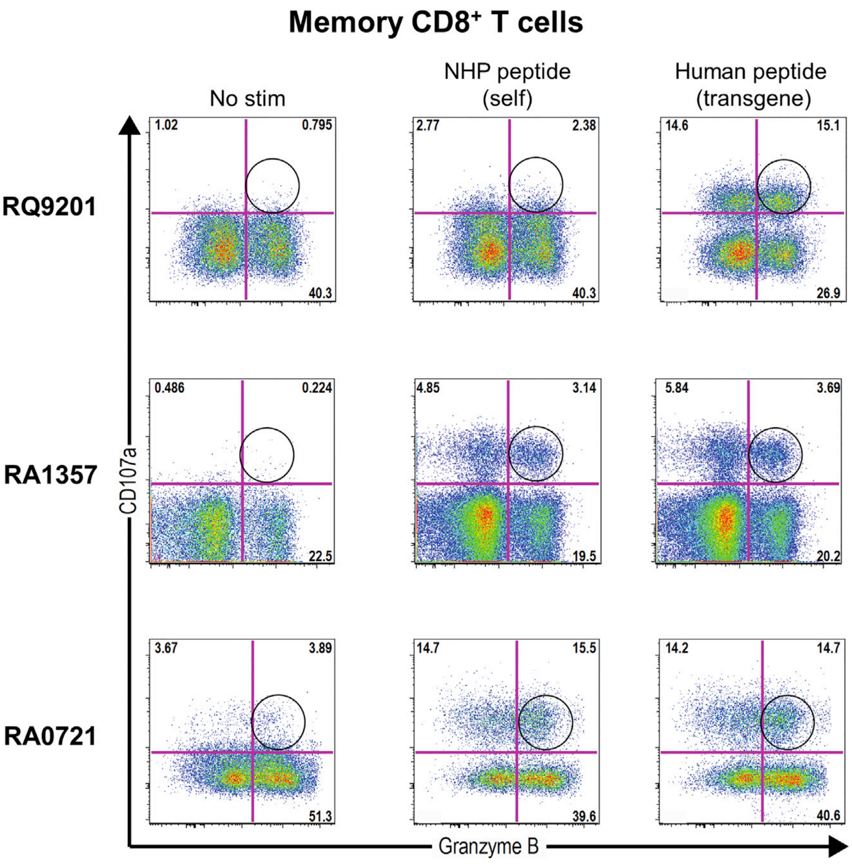

Figure 5. T Cell Responses to Macaque UGT1A1

Lymphocytes isolated from the spleens of animals RQ9201, RA1357, and RA0721 were stimulated with the human UGT1A1 immunodominant epitope (HAGKILLIPV), the NHP UGT1A1 epitope (HAGKMLLIPV), or dimethyl sulfoxide (No stim). Surface expression of a degranulation factor (CD107a) and expression of granzyme B by memory $CD8^+$ T cells were analyzed by ICS assay. Memory T cells were defined as $CD95^+$, $CD28^+$ and $CD95^+$, $CD28^−$. Circles indicate the presence of $CD107a^+$, granzyme B^+ UGT1A1-specific T cells.

response to both human and macaque UGT1A1, thus breaking tolerance to the self-antigen. MHC analysis of all animals enrolled in this study revealed a diverse collection of MHC haplotypes, even in macaques that developed a cross-reactive response to UGT1A1. We could not associate a specific MHC haplotype to a cross-reactive response to macaque UGT1A1. Interestingly, liver toxicity, ALT and AST levels, and the lack of major liver infiltrations were similar among these three animals.

We also observed that the AAV8.TBG.hUGT1A1co vector elicited a strong response to the hUGT1A1 transgene found in tissue-resident T cells, but not in blood. Flow cytometry analysis of these tissue-resident T cells revealed that the response was dominated by $CD4^+$ and $CD8^+$ T cells that are capable of degranulating ($CD107a^+$, granzyme B^+) but with minimal or no cytokine release. Tissue-resident memory T cells have been shown to elicit strong antigen-specific degranulating responses in animal models.[24] Degranulation of granzyme B has been associated with a mechanism that allows the killing of antigen-presenting cells by $CD4^+$ regulatory T cells,[25,26] therefore impairing an effective cytotoxic immune response by $CD8^+$ T cells. It is important to note the magnitude of the $CD107^+$ T cell responses in tissues (yielding up to 68% $CD4^+$ T cell response in one animal). *In vivo* studies have shown that this regulatory T cell response is induced and localized in secondary lymphoid tissues.[27] Although we did not test similar destruction of antigen-presenting cells, the data raise the possibility that $CD4^+$ and $CD8^+$ T cells possess tolerization potential under certain conditions. This interpretation is supported by the lack of observed toxicity in our animals, even in the presence of a large population of UGT1A1-specific $CD8^+$ T cells in the liver.

The potential for $CD8^+$ T cells to contribute to transgene tolerization and the liver-restricted biodistribution of transgene-specific T cells bears relevance for clinical translation of AAV gene therapies. Typical ELISPOT analyses in gene therapy clinical trials rely on detection of secreted IFN-γ in PBMCs. Significant fractions of the transgene-specific T cells observed here did not secrete activating cytokines, and were only observed in cells isolated from liver and not in cells isolated from peripheral blood. ICS analysis revealed that tissue-resident T cells expressed high levels of the homing and residency markers CXCR6 and CD69, which we would expect to prevent the escape of the cells from the liver into the circulating blood. AAV8 vectors mainly transduce the liver.[18,28–30] Therefore, UGT1A1 gene expression mainly occurs in the liver. Kupffer cells can prime T cells in the liver;[31] these T cells can then expand and exert their function within the liver. The expression of CD69 and CXCR6 would impede T cells from leaving the liver. As a consequence, UGT1A1-specific T cells would be undetectable in blood. This mechanism explains why T cell responses in peripheral blood were not correlated with T cell responses in tissues, suggesting that the continued monitoring of PBMCs during the in-life phase of studies and clinical trials is not a strong predictor of potential reactions in tissues.

In summary, the results of this study support the clinical translation of AAV8.TBG.hUGT1A1co for treatment of Crigler-Najjar syndrome. i.v. administration of a single dose of AAV8.TBG.hUGT1A1co vector in rhesus macaques effectively transduced and expressed the hUGT1A1 transgene in up to 37% of hepatocytes without leading to major liver toxicity. Importantly, there was no evidence of innate immune activation or systemic inflammation. Interestingly, administering the vector triggered a strong T cell response to UGT1A1, but this response was mainly restricted to tissue-resident

Table 1. Summary of NHPs

NHP ID	Sex	Dose (GC/kg)	Mamu Class I	Mamu Class II
RQ9201	female	2.5×10^{13}	A*04:/; B*03a:69	DRB*40:43; DQA*01_08:05_02; DBQ*06_07:/; DPA*02 g2:04 g2; DPB*07_03:02_01
RA1260	male	2.5×10^{13}	A*04:12; B*56d:18	DRB*52d:52d; DQA*01 g1:01 g3; DBQ*06 g3:06 g1; DPA*07_w_01:08 g; DPB*/:07_01
RA1261	male	2.5×10^{13}	A*01:19; B*17c:90	DRB*13c:22; DQA*01 g1:24 g2; DBQ*06 g3:15_02; DPA*02_w_05:08 g; DPB*01_w_03:04 g
RA0721	female	1.0×10^{13}	A*18b:49; B69b:/	DRB*13d:43; DQA*01_08:24 g2; DBQ*06_07:15_02; DPA*02_w_05:/; DPB*01_w_03:07_03
RA1357	male	1.0×10^{13}	A*32:51; B*69a:39a	DRB*nov9:50; DQA*01 g2:05_02; DBQ*06 g2:/; DPA*02_w_05:02 g1; DPB*01_w_03:15 g2
RA1846	male	1.0×10^{13}	A*48a:19; B*56d:68	DRB*03f:41; DQA*01_06:01_06; DBQ*06_13:06_15; DPA*06 g:07_w_04; DPB*01 g1_:/
RA0078	female	0	A*65b:73; B*15a:69a	DRB*58:06b; DQA*23_01:26 g3; DBQ*18_02:18_15; DPA*02 g1:04_02; DPB*15 g2:02_03
RA1274	male	0	A*701:12; B*86a:39a	DRB*16f:50; DQA*23_02:05_02; DBQ*18 g2:/: DPA*02 g1:02 g1; DPB*01_08:15 g2

T cells and not peripheral blood T cells. Furthermore, this response did not lead to liver toxicity. This study underscores the importance of studying immune responses at the site of AAV vector transduction and highlights the limitations of using blood as a surrogate to evaluate tissue-restricted T cell responses.

MATERIALS AND METHODS

Vector Production

AAV vectors expressing hUGT1A1co from the TBG promoter were produced by the Penn Vector Core at the University of Pennsylvania, as described previously.[32]

Animal Experimentation and Vector Administration

All animal procedures were performed in accordance with protocols approved by the Institutional Animal Care and Use Committee of the University of Pennsylvania.

Wild-type rhesus macaques that were 2–6 years old (n = 8) were obtained from Covance (Princeton, NJ). NHP studies were conducted at the University of Pennsylvania within a facility that is United States Department of Agriculture-registered, Association for Assessment and Accreditation of Laboratory Animal Care-accredited, and Public Health Service-assured. As previously described,[33] animals were housed in stainless steel cages with perches. All cage sizes and housing conditions were in compliance with the Guide for the Care and Use of Laboratory Animals. A 12-hr light-dark cycle was maintained and controlled via an Edstrom Watchdog system (Waterford, WI). Animals were fed Certified Primate Diet 5048 (PMI Feeds, Brentwood, MO) two times per day (morning and evening). An additional variety of food treats that were fit for human consumption, including fruits, vegetables, nuts, and cereals, were given daily as part of the standard enrichment process. Manipulanda such as kongs, mirrors, a puzzle feeder, and raisin balls were provided daily. Animals also received visual enrichment along with human interaction on a daily basis. All interventions were performed during the light cycle, and animals were fasted overnight prior to being anesthetized.

On study day 0, macaques were administered with 10 mL of vector or vehicle control into a peripheral vein at a rate of 1 mL/min via an infusion pump (Harvard Apparatus, Holliston, MA). This study complied with good laboratory practices for non-clinical laboratory studies with the following exceptions: the liver biopsy procedure, AAV8 NAb assay, IFN-γ ELISPOT, ISH analysis, vector GC and transgene RNA analysis, and immunological characterization of the immune response to UGT1A1. In addition, the evaluation of fibrin degradation products were not run under good laboratory practices conditions at Antech GLP (Morrisville, NC).

Analyses during the In-Life Phase

Rhesus macaques were anesthetized, and blood was collected on days −16, −7, 0, 8, 14, 21, 28, 31, 35, 42, 49, and 56 via the femoral vein for analysis of complete blood counts, clinical chemistries, and coagulation panels by Antech GLP (Morrisville, NC). NAb titers were determined on serum samples taken prior to initiation of the study, as described previously.[15] PBMCs were isolated and cryopreserved. T cell responses to AAV8 and hUGT1A1 were analyzed by IFN-γ ELISPOT, as previously described,[34] using peptide libraries specific for the AAV8 capsid and hUGT1A1 transgene; positive response criteria were >55 spot-forming units per 10^6 cells and three times the medium negative control value upon no stimulation. Positive responses in the IFN-γ ELISPOT assay were further analyzed, as previously described,[35] to determine the specific amino acid sequence (i.e., epitope) responsible for the T cell activation.

Liver Biopsy

On day 28 post-vector-administration, a laparotomy procedure was performed to isolate liver tissue. Collected samples were divided into those for histopathology (i.e., fixed in 10% neutral buffered

formalin) and biodistribution (i.e., frozen on dry ice and stored at −60°C or colder).

Necropsy and Analysis of Tissues for Pathology and Immunotoxicity

At day 56 post-vector-administration, rhesus macaques were euthanized and necropsied; tissues were harvested for full pathology. All tissues collected for histopathology were fixed using 10% neutral-buffered formalin, paraffin embedded, sectioned, and stained with H&E stain (Table S1). Slides were examined microscopically by a board-certified veterinary pathologist in a blinded manner.

In addition, lymphocytes were isolated from liver, spleen, and bone marrow, as described previously.[28] T cell responses to AAV8 and hUGT1A1 were analyzed by IFN-γ ELISPOT, in which the positive response criteria were the same as described above.

Molecular Analyses of Tissues

Tissues collected for biodistribution at liver biopsy and necropsy time points were frozen on dry ice. DNA and RNA was extracted from liver, and TaqMan qPCR reactions were performed, as previously described.[33,36]

ISH

Liver samples were fixed in 10% neutral buffered formalin and used for determination of hUGT1A1co messenger RNA expression by ISH, as described previously.[21] Z-shaped probe pairs specific for hUGT1A1co were synthesized by the kit manufacturer. Sections were counterstained with DAPI to show nuclei. To quantify RNA expression, five random pictures were taken from each liver section. Using ImageJ software (Rasband W.S., NIH, Bethesda, MD; https://imagej.nih.gov/ij/), a threshold was determined to select a UGT1A1-ISH-positive area, and the percentage of positive area per image area was established. Likewise, "empty" liver area (i.e., veins) was quantified in a second measurement. The final percentage of ISH-positive liver tissue (i.e., the percentage of positive hepatocytes) was then calculated per adjusted area (i.e., total area minus empty area), and the values were averaged for each liver sample.

MHC Typing

MHC typing of the rhesus macaques in this study was performed by Dr. David O'Connor at the University of Wisconsin-Madison using Fluidigm/MiSeq MHC genotyping assays (South San Francisco, CA), as previously described.[37]

ICS

The following antibodies were used: CD69 phycoerythrin (PE) (clone FN50), CD95 PE Cy5 (clone DX2), CD3 allophycocyanin (APC) Cy7 (clone SP34-2), and granzyme B AF700 (clone GB11) from BD Biosciences (San Diego, CA); IL-2 BV421 (clone MQ1-17H12), CD8a BV570 (clone RPA-T8), CCR7 BV711 (clone G043H7), CD14 BV650 (clone M5E2), CD20 BV650 (clone 2H7), CD16 BV650 (clone 3G8), IFN-γ (clone 4S.B3), TNF-α BV605 (clone Mab11), CD107a PE Cy7 (clone H4A3), and goat anti-mouse immunoglobulin G (IgG) AF488 (polyclonal, Poly4053) from Biolegend (San Diego, CA); CD28 R PE-Texas Red-X (ECD) (clone CD28.2) from Beckman Coulter (Indianapolis, IN); and CD4 PE Cy5.5 (clone S3.5) from Invitrogen (Carlsbad, CA). A Live/Dead Fixable Aqua Dead Cell Stain Kit (Invitrogen, Carlsbad, CA) was used for viability exclusion. Anti-CXCR6 antibody (clone 20D8) was kindly provided by Dr. Ronald G. Collman at the University of Pennsylvania.

Cells were thawed and resuspended at 2 million cells/mL in RPMI medium with 10% fetal bovine serum, 2 mM L-glutamine, 100 U/mL penicillin, and 100 mg/mL streptomycin; they were rested overnight in a 37°C, 5% CO_2 incubator. *In vitro* stimulations were performed the following morning. In brief, cells were transferred to a 96-well V-bottom plate at 1–2 million cells/well and divided into three conditions: stimulated with peptide pools at a final concentration of 2 μg/mL, unstimulated control (only dimethyl sulfoxide, no peptides), or phorbol 12-myristate 13-acetate (100 ng/μL) and ionomycin (1 μg/mL). Cells were incubated for 1 hr at 37°C in the presence of CD49d stimulatory monoclonal antibody (mAb) (clone 9F10, Biolegend, San Diego, CA) and CD107a, followed by an additional 8-hr incubation with monensin (0.7 μg/mL final concentration; BD Biosciences, San Diego, CA) and brefeldin A (1 μg/mL final concentration; Sigma-Aldrich, St. Louis, MO), as previously described.[38] Cells were washed in PBS and stained with anti-CXCR6 for 25 min at 37°C, 5% CO_2, washed again in PBS, and then secondary stained with goat anti-mouse IgG AF488 for an additional 25 min at 37°C, 5% CO_2. Anti-CCR7 was then added and incubated for 15 min at 37°C, 5% CO_2. All of the following staining procedures were performed at room temperature in the dark. Cells were washed once with PBS and stained for viability with Aqua amine-reactive dye (Invitrogen, Carlsbad, CA) for 10 min. The extracellular antibody cocktail prepared in PBS with 0.1% sodium azide and 1% BSA was added and incubated for 15 min. Cells were then washed and permeabilized with the BD Cytofix/Cytoperm Kit (BD Biosciences, San Diego CA; catalog 554722) according to manufacturer's instructions. Intracellular antibody cocktail was incubated for 1 hr. Following intracellular staining, cells were washed and fixed with PBS with 1% paraformaldehyde and stored in the dark until acquisition. All flow cytometry data were collected on an LSR D cytometer (BD Biosciences, San Diego, CA). Data were analyzed using FlowJo software (Tree Star, Ashland, OR).

Statistical Analyses

All values are expressed as mean ± SEM unless otherwise stated. Differences in ALT and AST levels compared to baseline levels were analyzed statistically by the Wilcoxon rank-sum test. Overall differences in ALT and AST values across all time points were analyzed using a linear mixed-effect model. A p value of <0.05 was considered significant. Due to the low number in the control group, further statistical analyses could not be performed.

SUPPLEMENTAL INFORMATION

Supplemental Information includes five figures and one table and can be found with this article online at https://doi.org/10.1016/j.omtm.2018.10.012.

AUTHOR CONTRIBUTIONS

J.A.G., R.C., L.K.-C., M.R.B., and J.M.W. conceived and designed the experiments; J.A.G., R.C., L.K.-C., J.M.L.N., J.A., E.B., and T.G. performed the experiments; J.A.G., R.C., L.K.-C., E.A.C., P.B., L.K.R., M.R.B., and J.M.W. analyzed the data; and J.A.G., R.C., L.K.-C., M.R.B., and J.M.W. contributed to the writing of the manuscript.

CONFLICTS OF INTEREST

J.M.W. is an advisor to, holds equity in, and has a sponsored research agreement with REGENXBIO and Scout Bio; he also has a sponsored research agreement with Ultragenyx, Biogen, and Janssen, which are licensees of Penn technology. In addition, he has sponsored research agreements with Precision Biosciences, Moderna Therapeutics, and Amicus Therapeutics. J.M.W. is an inventor on patents that have been licensed to various biopharmaceutical companies.

ACKNOWLEDGMENTS

This work was funded by Audentes Therapeutics. We thank Mingyao Li and Yan Che for statistical analysis; Shu-Jen Chen and Michael Korn for shedding and biodistribution studies; and John T. Gray for providing helpful comments on the manuscript. Invaluable technical assistance was provided by the Nonhuman Primate Research Program, the Immunology Core, Vector Clinical Services, and the Morphology Core of the Gene Therapy Program of the University of Pennsylvania. We also thank Dr. Katherine S. Wetzel and Dr. Ronald G. Collman from the University of Pennsylvania for providing the CXCR6 antibody used for ICS.

REFERENCES

1. Kassim, S.H., Li, H., Bell, P., Somanathan, S., Lagor, W., Jacobs, F., Billheimer, J., Wilson, J.M., and Rader, D.J. (2013). Adeno-associated virus serotype 8 gene therapy leads to significant lowering of plasma cholesterol levels in humanized mouse models of homozygous and heterozygous familial hypercholesterolemia. Hum. Gene Ther. *24*, 19–26.

2. Greig, J.A., Limberis, M.P., Bell, P., Chen, S.J., Calcedo, R., Rader, D.J., and Wilson, J.M. (2017). Non-Clinical Study Examining AAV8.TBG.hLDLR Vector-Associated Toxicity in Chow-Fed Wild-Type and LDLR$^{+/-}$ Rhesus Macaques. Hum. Gene Ther. Clin. Dev. *28*, 39–50.

3. Greig, J.A., Limberis, M.P., Bell, P., Chen, S.J., Calcedo, R., Rader, D.J., and Wilson, J.M. (2017). Nonclinical Pharmacology/Toxicology Study of AAV8.TBG.mLDLR and AAV8.TBG.hLDLR in a Mouse Model of Homozygous Familial Hypercholesterolemia. Hum. Gene Ther. Clin. Dev. *28*, 28–38.

4. Wang, L., Wang, H., Morizono, H., Bell, P., Jones, D., Lin, J., McMenamin, D., Yu, H., Batshaw, M.L., and Wilson, J.M. (2012). Sustained correction of OTC deficiency in spf(ash) mice using optimized self-complementary AAV2/8 vectors. Gene Ther. *19*, 404–410.

5. Sarkar, R., Tetreault, R., Gao, G., Wang, L., Bell, P., Chandler, R., Wilson, J.M., and Kazazian, H.H., Jr. (2004). Total correction of hemophilia A mice with canine FVIII using an AAV 8 serotype. Blood *103*, 1253–1260.

6. Wang, L., Calcedo, R., Nichols, T.C., Bellinger, D.A., Dillow, A., Verma, I.M., and Wilson, J.M. (2005). Sustained correction of disease in naive and AAV2-pretreated hemophilia B dogs: AAV2/8-mediated, liver-directed gene therapy. Blood *105*, 3079–3086.

7. Greig, J.A., Wang, Q., Reicherter, A.L., Chen, S.J., Hanlon, A.L., Tipper, C.H., Clark, K.R., Wadsworth, S., Wang, L., and Wilson, J.M. (2017). Characterization of Adeno-Associated Viral Vector-Mediated Human Factor VIII Gene Therapy in Hemophilia A Mice. Hum. Gene Ther. *28*, 392–402.

8. Greig, J.A., Nordin, J.M.L., Draper, C., McMenamin, D., Chroscinski, E.A., Bell, P., Gray, J.T., Richman, L.K., and Wilson, J.M. (2018). Determining the Minimally Effective Dose of a Clinical Candidate AAV Vector in a Mouse Model of Crigler-Najjar Syndrome. Mol. Ther. Methods Clin. Dev. *10*, 237–244.

9. Wakim, L.M., Waithman, J., van Rooijen, N., Heath, W.R., and Carbone, F.R. (2008). Dendritic cell-induced memory T cell activation in nonlymphoid tissues. Science *319*, 198–202.

10. Gebhardt, T., Wakim, L.M., Eidsmo, L., Reading, P.C., Heath, W.R., and Carbone, F.R. (2009). Memory T cells in nonlymphoid tissue that provide enhanced local immunity during infection with herpes simplex virus. Nat. Immunol. *10*, 524–530.

11. Masopust, D., Choo, D., Vezys, V., Wherry, E.J., Duraiswamy, J., Akondy, R., Wang, J., Casey, K.A., Barber, D.L., Kawamura, K.S., et al. (2010). Dynamic T cell migration program provides resident memory within intestinal epithelium. J. Exp. Med. *207*, 553–564.

12. Masopust, D., Vezys, V., Marzo, A.L., and Lefrançois, L. (2014). Pillars article: preferential localization of effector memory cells in nonlymphoid tissue. Science. 2001. 291: 2413-2417. J. Immunol. *192*, 845–849.

13. Mackay, L.K., Rahimpour, A., Ma, J.Z., Collins, N., Stock, A.T., Hafon, M.L., Vega-Ramos, J., Lauzurica, P., Mueller, S.N., Stefanovic, T., et al. (2013). The developmental pathway for CD103(+)CD8+ tissue-resident memory T cells of skin. Nat. Immunol. *14*, 1294–1301.

14. Buggert, M., Nguyen, S., Salgado-Montes de Oca, G., Bengsch, B., Darko, S., Ransier, A., Roberts, E.R., Del Alcazar, D., Brody, I.B., Vella, L.A., et al. (2018). Identification and characterization of Hi.v.-specific resident memory CD8^{+} T cells in human lymphoid tissue. Sci. Immunol. *3*, eaar4526.

15. Calcedo, R., Vandenberghe, L.H., Gao, G., Lin, J., and Wilson, J.M. (2009). Worldwide epidemiology of neutralizing antibodies to adeno-associated viruses. J. Infect. Dis. *199*, 381–390.

16. Calcedo, R., and Wilson, J.M. (2016). AAV Natural Infection Induces Broad Cross-Neutralizing Antibody Responses to Multiple AAV Serotypes in Chimpanzees. Hum. Gene Ther. Clin. Dev. *27*, 79–82.

17. Harding, T.C., Koprivnikar, K.E., Tu, G.H., Zayek, N., Lew, S., Subramanian, A., Sivakumaran, A., Frey, D., Ho, K., VanRoey, M.J., et al. (2004). Intravenous administration of an AAV-2 vector for the expression of factor IX in mice and a dog model of hemophilia B. Gene Ther. *11*, 204–213.

18. Gao, G., Lu, Y., Calcedo, R., Grant, R.L., Bell, P., Wang, L., Figueredo, J., Lock, M., and Wilson, J.M. (2006). Biology of AAV serotype vectors in liver-directed gene transfer to nonhuman primates. Mol. Ther. *13*, 77–87.

19. Hurlbut, G.D., Ziegler, R.J., Nietupski, J.B., Foley, J.W., Woodworth, L.A., Meyers, E., Bercury, S.D., Pande, N.N., Souza, D.W., Bree, M.P., et al. (2010). Preexisting immunity and low expression in primates highlight translational challenges for liver-directed AAV8-mediated gene therapy. Mol. Ther. *18*, 1983–1994.

20. Greig, J.A., Nordin, J.M., Bote, E., Makaron, L., Garnett, M.E., Kattenhorn, L.M., Bell, P., Goode, T., and Wilson, J.M. (2016). Impact of intravenous infusion time on AAV8 vector pharmacokinetics, safety, and liver transduction in cynomolgus macaques. Mol. Ther. Methods Clin. Dev. *3*, 16079.

21. Hinderer, C., Katz, N., Buza, E.L., Dyer, C., Goode, T., Bell, P., Richman, L.K., and Wilson, J.M. (2018). Severe Toxicity in Nonhuman Primates and Piglets Following High-Dose Intravenous Administration of an Adeno-Associated Virus Vector Expressing Human SMN. Hum. Gene Ther. *29*, 285–298.

22. Hordeaux, J., Wang, Q., Katz, N., Buza, E.L., Bell, P., and Wilson, J.M. (2018). The Neurotropic Properties of AAV-PHP.B Are Limited to C57BL/6J Mice. Mol. Ther. *26*, 664–668.

23. Greig, J.A., Nordin, J.M.L., Draper, C., Bell, P., and Wilson, J.M. (2018). AAV8 Gene Therapy Rescues the Newborn Phenotype of a Mouse Model of Crigler-Najjar. Hum. Gene Ther. *29*, 763–770.

24. Fernandez-Ruiz, D., Ng, W.Y., Holz, L.E., Ma, J.Z., Zaid, A., Wong, Y.C., Lau, L.S., Mollard, V., Cozijnsen, A., Collins, N., et al. (2016). Liver-Resident Memory CD8^{+} T Cells Form a Front-Line Defense against Malaria Liver-Stage Infection. Immunity *45*, 889–902.

25. Grossman, W.J., Verbsky, J.W., Tollefsen, B.L., Kemper, C., Atkinson, J.P., and Ley, T.J. (2004). Differential expression of granzymes A and B in human cytotoxic lymphocyte subsets and T regulatory cells. Blood *104*, 2840–2848.

26. Magnani, C.F., Alberigo, G., Bacchetta, R., Serafini, G., Andreani, M., Roncarolo, M.G., and Gregori, S. (2011). Killing of myeloid APCs via HLA class I, CD2 and CD226 defines a novel mechanism of suppression by human Tr1 cells. Eur. J. Immunol. *41*, 1652–1662.

27. Huber, S., Gagliani, N., Esplugues, E., O'Connor, W., Jr., Huber, F.J., Chaudhry, A., Kamanaka, M., Kobayashi, Y., Booth, C.J., Rudensky, A.Y., et al. (2011). Th17 cells express interleukin-10 receptor and are controlled by Foxp3− and Foxp3+ regulatory CD4+ T cells in an interleukin-10-dependent manner. Immunity *34*, 554–565.

28. Wang, L., Calcedo, R., Wang, H., Bell, P., Grant, R., Vandenberghe, L.H., Sanmiguel, J., Morizono, H., Batshaw, M.L., and Wilson, J.M. (2010). The pleiotropic effects of natural AAV infections on liver-directed gene transfer in macaques. Mol. Ther. *18*, 126–134.

29. Wang, L., Wang, H., Bell, P., McCarter, R.J., He, J., Calcedo, R., Vandenberghe, L.H., Morizono, H., Batshaw, M.L., and Wilson, J.M. (2010). Systematic evaluation of AAV vectors for liver directed gene transfer in murine models. Mol. Ther. *18*, 118–125.

30. Bell, P., Wang, L., Gao, G., Haskins, M.E., Tarantal, A.F., McCarter, R.J., Zhu, Y., Yu, H., and Wilson, J.M. (2011). Inverse zonation of hepatocyte transduction with AAV vectors between mice and non-human primates. Mol. Genet. Metab. *104*, 395–403.

31. Breous, E., Somanathan, S., Vandenberghe, L.H., and Wilson, J.M. (2009). Hepatic regulatory T cells and Kupffer cells are crucial mediators of systemic T cell tolerance to antigens targeting murine liver. Hepatology *50*, 612–621.

32. Lock, M., Alvira, M., Vandenberghe, L.H., Samanta, A., Toelen, J., Debyser, Z., and Wilson, J.M. (2010). Rapid, simple, and versatile manufacturing of recombinant adeno-associated viral vectors at scale. Hum. Gene Ther. *21*, 1259–1271.

33. Greig, J.A., Peng, H., Ohlstein, J., Medina-Jaszek, C.A., Ahonkhai, O., Mentzinger, A., Grant, R.L., Roy, S., Chen, S.J., Bell, P., et al. (2014). Intramuscular injection of AAV8 in mice and macaques is associated with substantial hepatic targeting and transgene expression. PLoS ONE *9*, e112268.

34. Calcedo, R., Vandenberghe, L.H., Roy, S., Somanathan, S., Wang, L., and Wilson, J.M. (2009). Host immune responses to chronic adenovirus infections in human and nonhuman primates. J. Virol. *83*, 2623–2631.

35. Calcedo, R., Somanathan, S., Qin, Q., Betts, M.R., Rech, A.J., Vonderheide, R.H., Mueller, C., Flotte, T.R., and Wilson, J.M. (2017). Class I-restricted T-cell responses to a polymorphic peptide in a gene therapy clinical trial for α-1-antitrypsin deficiency. Proc. Natl. Acad. Sci. USA *114*, 1655–1659.

36. Chen, S.-J., Sanmiguel, J., Lock, M., McMenamin, D., Draper, C., Limberis, M.P., Kassim, S.H., Somanathan, S., Bell, P., Johnston, J.C., et al. (2013). Biodistribution of AAV8 vectors expressing human low-density lipoprotein receptor in a mouse model of homozygous familial hypercholesterolemia. Hum. Gene Ther. Clin. Dev. *24*, 154–160.

37. Wiseman, R.W., Karl, J.A., Bimber, B.N., O'Leary, C.E., Lank, S.M., Tuscher, J.J., Detmer, A.M., Bouffard, P., Levenkova, N., Turcotte, C.L., et al. (2009). Major histocompatibility complex genotyping with massively parallel pyrosequencing. Nat. Med. *15*, 1322–1326.

38. Fukazawa, Y., Lum, R., Okoye, A.A., Park, H., Matsuda, K., Bae, J.Y., Hagen, S.I., Shoemaker, R., Deleage, C., Lucero, C., et al. (2015). B cell follicle sanctuary permits persistent productive simian immunodeficiency virus infection in elite controllers. Nat. Med. *21*, 132–139.

OMTM, Volume *11*

Supplemental Information

AAV8 Gene Therapy for Crigler-Najjar Syndrome in Macaques Elicited Transgene T Cell Responses That Are Resident to the Liver

Jenny A. Greig, Roberto Calcedo, Leticia Kuri-Cervantes, Jayme M.L. Nordin, Jessica Albrecht, Erin Bote, Tamara Goode, Edward A. Chroscinski, Peter Bell, Laura K. Richman, Michael R. Betts, and James M. Wilson

SUPPLEMENTAL INFORMATION

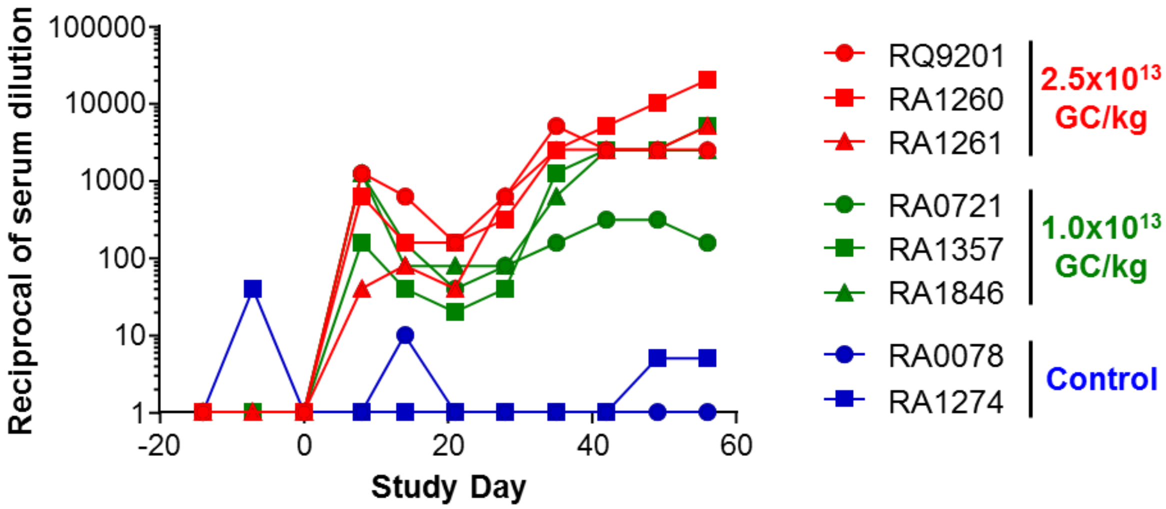

Figure S1. NAb levels in rhesus macaques administered with high or low dose of AAV8.TBG.hUGT1A1co compared to vehicle.
Rhesus macaques received an IV injection with 1.0x10^{13} GC/kg or 2.5x10^{13} GC/kg of AAV8.TBG.hUGT1A1co, or vehicle as a control. NAb levels to the AAV8 capsid were measured in serum samples taken throughout the study. The limit of detection of the assay is 5 (reciprocal of serum dilution).

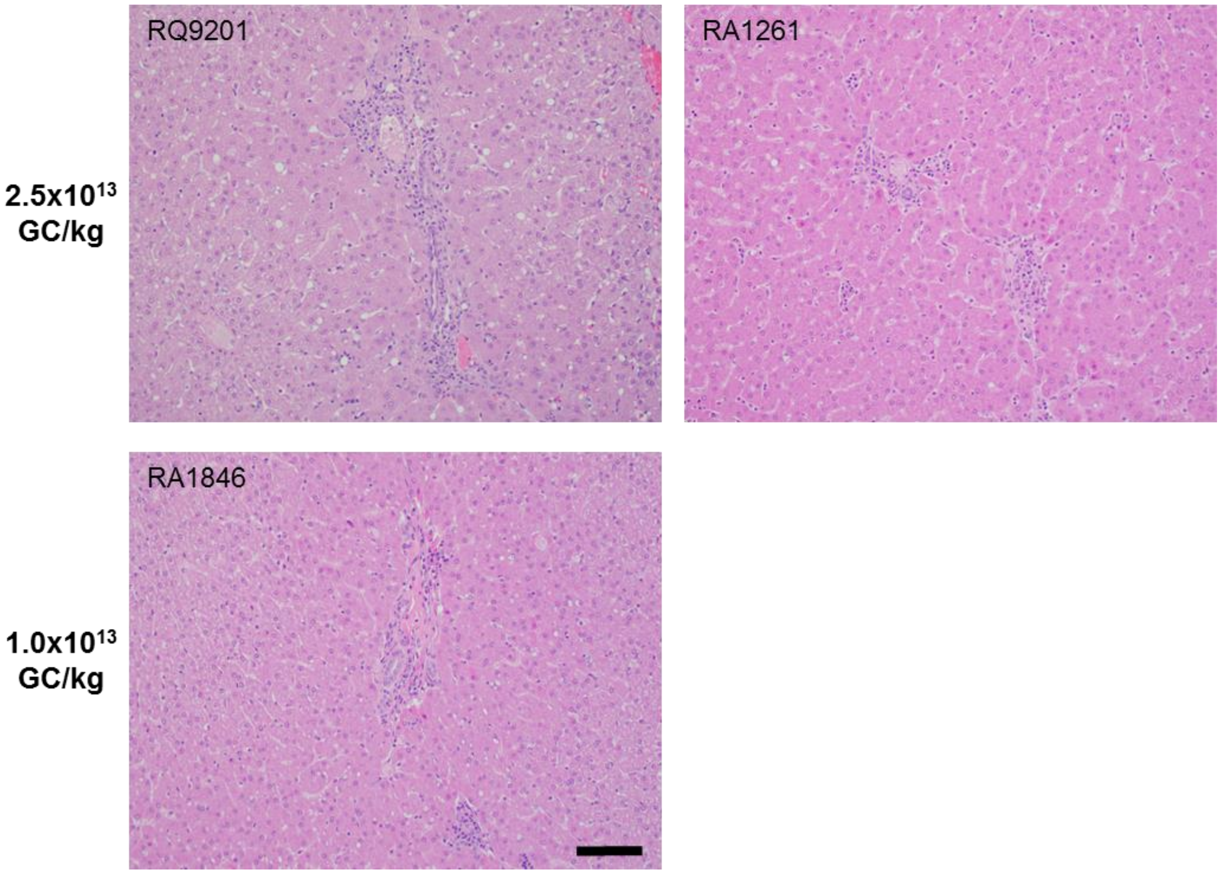

Figure S2. Liver histopathology in vector-administered macaques.
Representative images were taken from three macaques administered with vector to show areas with minimal to mild mononuclear cell infiltrate. Scale bar = 200 µm.

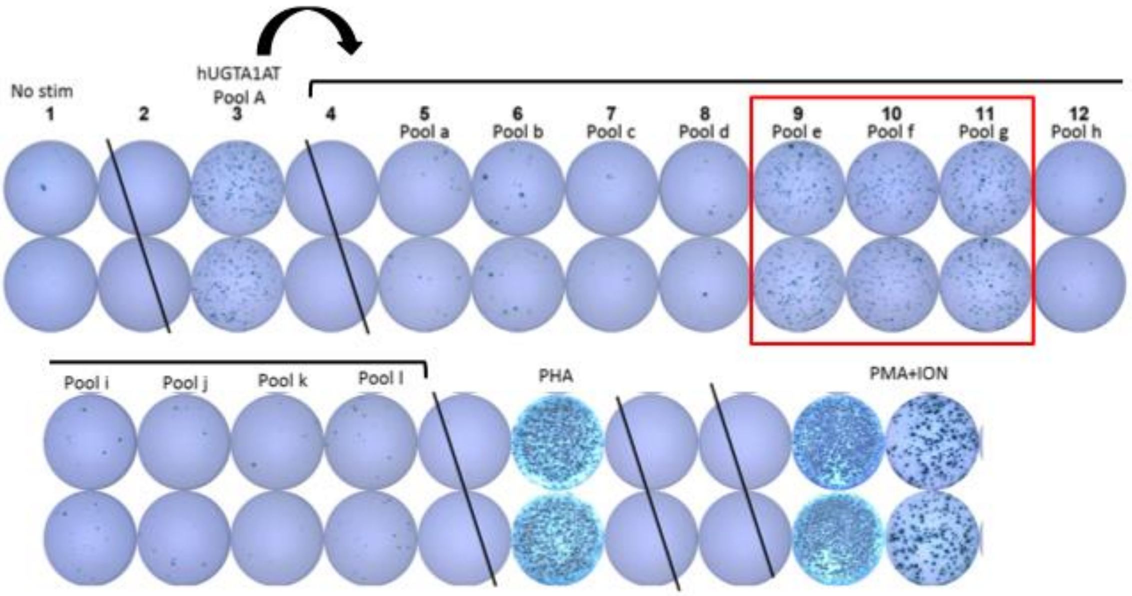

hUGT1A1 Pool A Sub-pools:

Pool	a	b	c	d	e	f
g	1	2	3	4	5	6
h	7	8	9	10	11	12
I	13	14	15	16	17	18
j	19	20	21	22	23	24
k	25	26	27	28	29	30
l	31	32	33	34	35	

5 : GPVVSHAGKILLIPV
6 : HAGKILLIPVDGSHW

Figure S3. Epitope mapping within the hUGT1A1 peptide pool A.
IFN-γ ELISPOT assay using PBMCs from animal RQ9201 that were stimulated with the hUGT1A1 peptide pool A and the matrix of sub-pools (a-l) generated from hUGT1A1 pool A. Positive responses were seen in sub-pools e, f, and g. The immunodominant epitope in the human sequence is shown in red. Phytohemagglutinin (PHA) or a phorbol 12-myristate 13-acetate and ionomycin (PMA+ION) at two cell concentration were used as stimuli for positive controls.

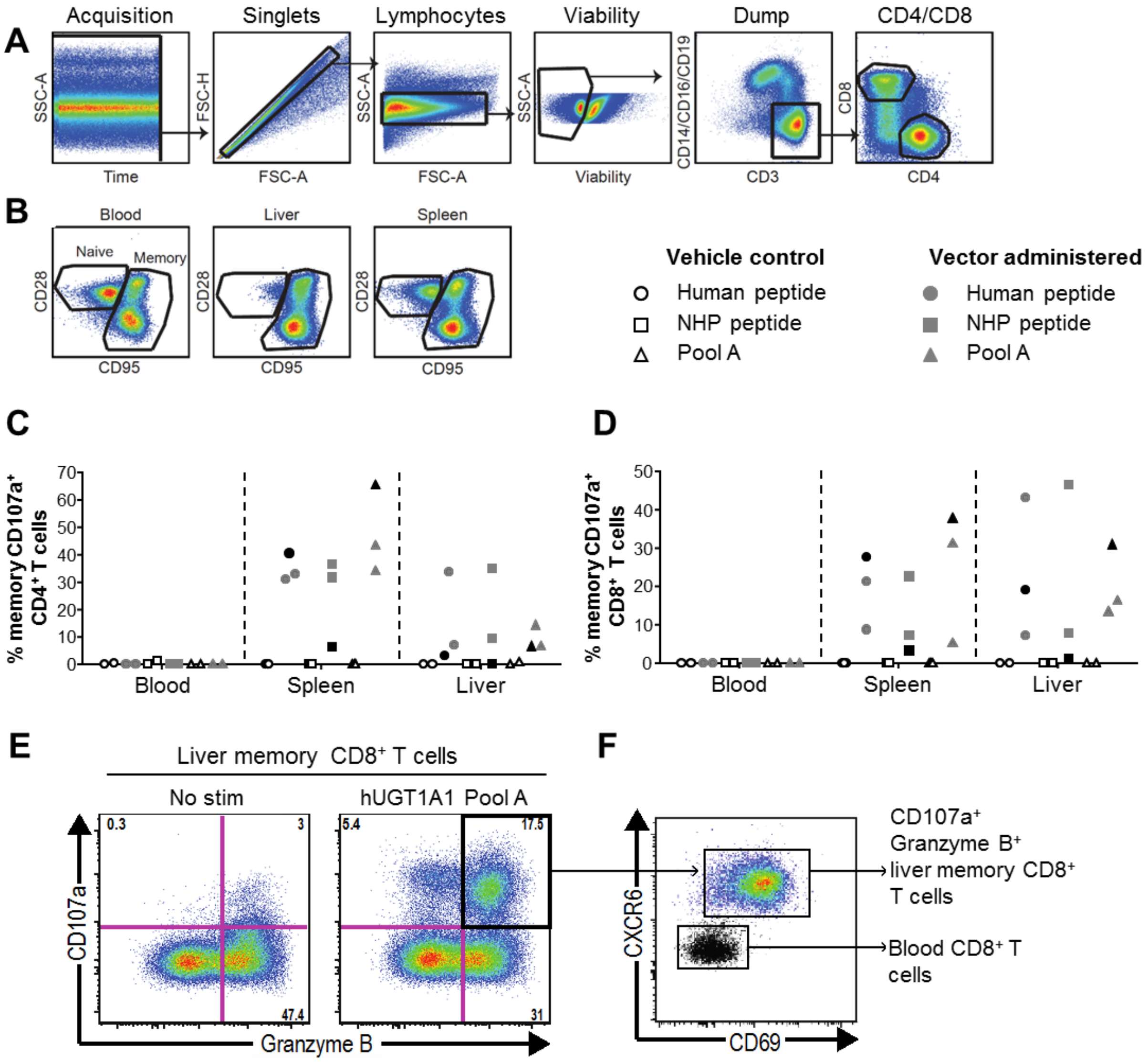

Figure S4. **Peptide-specific memory T-cell responses to AAV8.TBG.hUGT1A1co in blood and secondary lymphoid organs.**

(A) Representative plots of the gating strategy in peripheral blood where single viable lymphocytes were selected. $CD4^+$ or $CD8^+$ T cells were then selected from $CD3^+$ T cells, excluding monocytes, natural killer cells, and B cells. Naïve T cells ($CD28^+$, $CD95^-$) were gated out to further analyze peptide-specific responses within memory T cells. (B) Examples of the distribution of memory and naïve $CD8^+$ T cells in blood, liver, and spleen are shown. Lymphocytes from vehicle- (open symbols) or vector-administered rhesus macaques (closed symbols; low dose, gray; high dose, black) were cultured for nine hours in the presence of hUGT1A1 immunodominant epitope (circles, HAGKILLIPV), NHP UGT1A1 self-epitope (squares, HAGKMLLIPV), or hUGT1A1 pool A (triangles). Peptide-specific T-cell responses were measured in $CD107a^+$, $CD4^+$ memory T cells (C) and $CD107a^+$, $CD8^+$ memory T cells (D) derived from blood, spleen, and liver taken at the time of necropsy. (E) Representative plots of peptide-specific responses in liver lymphocytes of an animal administered with vector. The numbers inside the quadrants indicate percentages of memory $CD8^+$ T cells. (F) CXCR6/CD69 tissue-resident phenotype within peptide-specific $CD8^+$ T cells (color) in the liver and peripheral blood $CD8^+$ T cells (black).

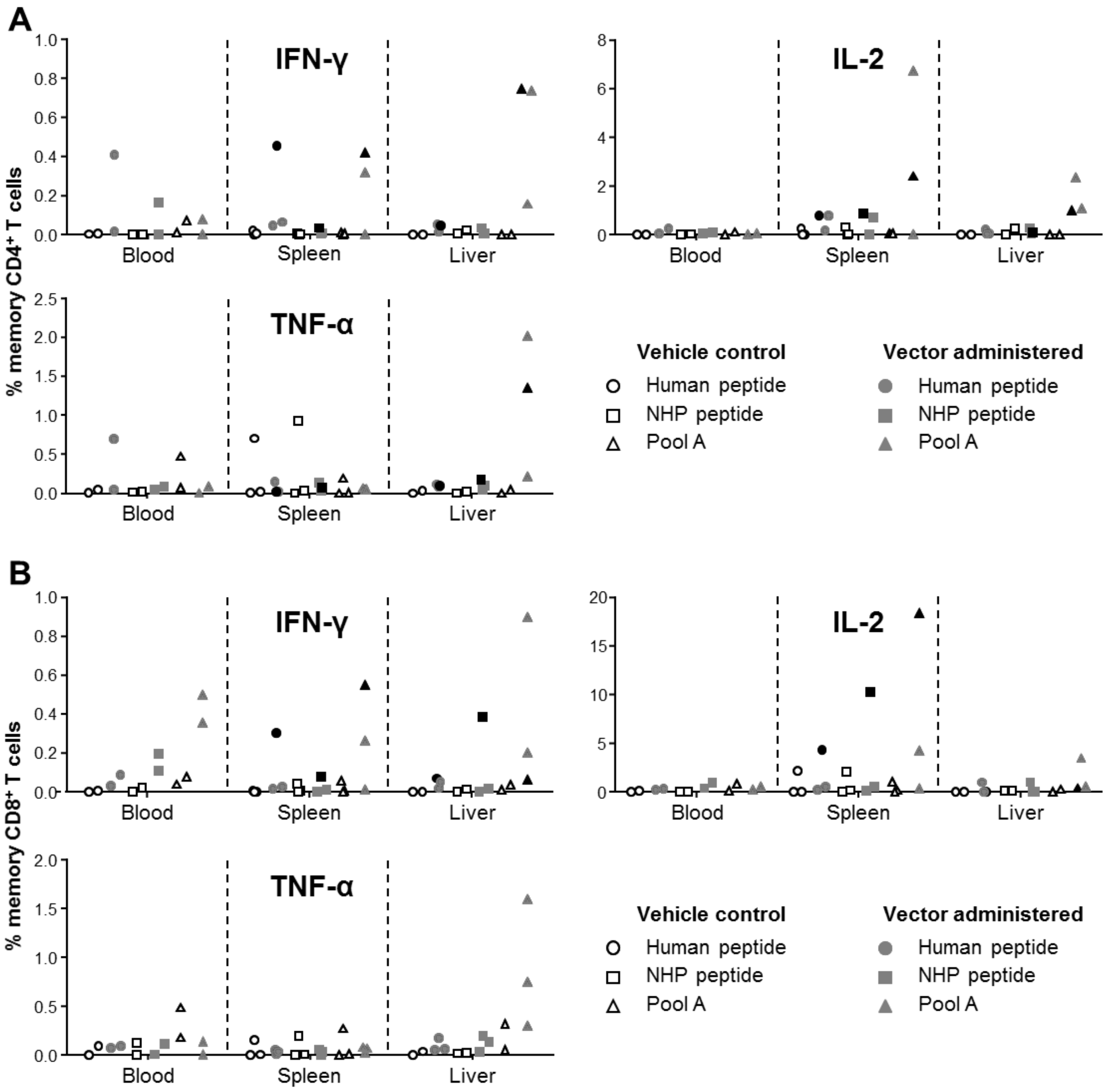

Figure S5. Flow cytometric analysis of lymphocytes isolated from peripheral blood and secondary lymphoid organs.

Peptide-specific production of IFN-γ, IL-2, and TNF-α in memory $CD4^+$ T cells (A) and $CD8^+$ T cells (B) from peripheral blood, spleen, and liver from vehicle- (open symbols) or vector-administered rhesus macaques (closed symbols; low dose, gray; high dose, black) were measured after nine hours of culture in response to stimulation with hUGT1A1 immunodominant epitope (circles, HAGKILLIPV), NHP UGT1A1 self-epitope (squares, HAGKMLLIPV), or hUGT1A1 pool A (triangles).

Tissues Collected For Histopathology

Adrenal gland, left	Liver (left, middle, right, caudate)	Small intestine, duodenum
Adrenal gland, right	Lung, left	Small intestine, jejunum
Ascending aorta (proximal)	Lung, right	Small intestine, ileum (Peyers Patch)
Bone marrow, rib	Lacrimal gland	Spinal Cord
Brain	Lymph node, mandibular	Spleen
Cecum	Lymph node, mesenteric	Stomach
Cervix	Muscle, quadriceps femoris	Testes
Epididimides	Ovaries	Urinary Bladder
Esophagus	Pancreas	Uterus
Eye (left)	Prostate	Pituitary
Gall bladder	Rectum	Thymus
Heart	Salivary gland, mandibular	Thyroid gland (with parathyroid)
Kidney, left	Sciatic nerves	Trachea
Kidney, right	Seminal Vesicle	Vagina
Large intestine, colon	Skin with mammary	Gross lesions (if any)

Table S1. Tissues collected for histopathology.
Tissues collected for histopathological analysis at necropsy.
